# Tic disorders revisited: introduction of the term “tic spectrum disorders”

**DOI:** 10.1007/s00787-018-01272-7

**Published:** 2019-01-19

**Authors:** Kirsten R. Müller-Vahl, Tanvi Sambrani, Ewgeni Jakubovski

**Affiliations:** 1 Clinic of Psychiatry, Social Psychiatry, and Psychotherapy, Hannover Medical School, Hannover, Germany; 2 Department of Education, Monash University, Melbourne, VIC Australia

**Keywords:** Tourette syndrome, Diagnosis, Nosology

## Abstract

Although the DSM-5 chronic motor tic disorder (CMTD) and Tourette syndrome (TS) are distinct diagnostic categories, there is no genetic or phenotypic evidence that supports this diagnostic categorization. The aim of this study was to compare patients with both diagnoses along a number of clinical characteristics to provide further diagnostic clarity. Our sample consisted of 1018 patients (including adult and child patients) suffering from chronic tic disorders. Tic severity was assessed via Shapiro Tourette-Syndrome Severity Scale (STSS). Lifetime prevalence of other comorbid conditions was assessed in a semi-structured clinical interview. The data were gained through retrospective chart analysis. The two groups did not differ significantly in any of the clinical or demographic variables. Patients only differed in tic severity, with CMTD patients (*n* = 40) having lower mean tic severity (STSS = 2.0 vs. 2.8; *p* < 0.001), prevalence of complex motor tics (27.5% vs. 55.9%; *p* < 0.01), copropraxia (0% vs. 16.2%; *p* < 0.01) and echopraxia (10.0% vs. 23.8%; *p* < 0.05), and a markedly lower comorbidity score (1.9 vs. 2.7; *p* < 0.001) as compared to TS patients (*n* = 978). Our results suggest that both disorders exist along a symptom severity continuum of which TS constitutes a more severe and CMTD a less severe form. We therefore suggest the introduction of the term “tic spectrum disorders”, instead of using different diagnostic categories.

**Electronic supplementary material:**

The online version of this article (10.1007/s00787-018-01272-7) contains supplementary material, which is available to authorized users.

## Introduction

Primary tic disorders are a group of childhood-onset neuropsychiatric disorders that are defined by the presence of one or more motor and/or phonic (= vocal) tics for a time period of less or more than 1 year. Tics typically have a waxing and waning course. Chronic tic disorders (defined by a duration > 1 year) are often associated with several other symptoms and comorbid diagnoses such as obsessive–compulsive behavior (OCB)/obsessive–compulsive disorder (OCD), attention deficit/hyperactivity disorder (ADHD), rage attacks, self-injurious behavior (SIB), as well as various mood and anxiety disorders [1]. Although well accepted, these comorbid conditions are not part of existing classifications for tic disorders. According to DSM-5 [2] and ICD-10 [3], primary tic disorders include Tourette syndrome (TS) (= combined phonic and motor tic disorder), persistent (or chronic) motor tic disorder (CMTD), persistent (or chronic) phonic tic disorder (CPTD), and provisional (or transient) tic disorder (PTD). Primary tic disorders are much more common than secondary tic disorders [4], which are caused by other conditions such as certain neurodegenerative disorders [5], stroke [6], or substances [7].

According to existing classifications, TS varies from CMTD in only the following way: in TS, it is required for both, multiple motor tics and at least one phonic tic be present, while in CMTD it is only required for motor tics to be present [2, 3]. Other characteristics such as number, severity, or complexity of the motor tics, or the kind/number of comorbidities are not used to differentiate between TS and CMTD in the DSM/ICD.

Several recent research studies have estimated that the prevalence rate of TS in children in the general population ranges from 0.3 to 0.9% [8–11], while the prevalence rate of CMTD ranges from 0.5 to 1.65% [8–11]. Due to this additional criterion regarding the added presence of phonic tics, TS in the community is rarer in comparison to CMTD [12, 13]. Accordingly, the prevalence for PTD—the mildest form of all primary tic disorders—is much higher, ranging from 5 up to 47% (for review see [14]).

Although there was a discussion during the preparation of DSM-5 as to whether or not a distinction should be made between TS and CMTD, the findings obtained from a few available studies highlighting various differences between the two conditions proved to be a compelling factor in favor of making TS and CMTD two separate disorders. For example, Diniz et al. [15] found phenotypic differences of tic symptoms among people with OCD, influenced by the nature of the tic disorder [i.e., TS or chronic tic disorder (CTD)]. These included an earlier age of onset of OCB, experiencing sensory phenomena before engaging in repetitive behavior, and presence of bipolar disorder among OCD + TS patients compared to OCD + CMTD patients [15].

On the other hand, there is emerging literature suggesting that both TS and CMTD are a part of the same clinical entity, with CMTD being a milder form of TS. Spencer et al. [13] found that patients with TS and those with CMTD are similar on several clinical correlates including impairments and kind of psychiatric comorbidities. Moreover, there is no evidence that the effect of treatment (medical or behavioral) for tics is different in CMTD compared to TS [16]. Additionally, to the best of our knowledge, there is no genetic or imaging study conducted so far that supports the idea that TS and CMTD have different underlying causes.

However, more research is required into exploring the similarities and differences between TS and CMTD, and therefore also looking at the many implications the nature of this relationship may have, specifically on their distinction in the DSM/ICD.

Through this study, we aim to explore whether there are other differences between TS and CMTD (besides the presence or absence of phonic tics in the former) that justify two different diagnoses. We hypothesized that there are no such further differences, and that TS and CMTD are part of the same spectrum in which TS is, on average, only a more severe form of CMTD.

## Method

The sample (*N* = 1018) consisted of both adult and child patients suffering from different primary tic disorders who had visited the Hannover Medical School (MHH), Germany—the largest TS center in the country—for a referral and had been attended to and diagnosed by one of the authors (KMV), who is an adult psychiatrist with an expertise in TS, as well as a neurologist. The diagnoses of both TS and CMTD were made based on DSM criteria valid during that period of time (1995–2015).

For each patient, lifetime data for simple motor and phonic tics, complex motor and phonic tics including specifically coprolalia, copropraxia, echolalia, echopraxia, and palilalia were obtained, and each symptom was scored as either present or absent. The Shapiro Tourette-Syndrome Severity Scale (STSS) was used to assess current tic severity on the day of visit at MHH. The STSS has five variables with matching rating scales, which are as follows: (1) tics noticeable to others (0–3), (2) tics that elicit comments or curiosity (0–1), (3) patients considered odd or bizarre (0–2), (4) tics that interfere with functioning (0–2), (5) patient incapacitated, homebound, or hospitalized (0–1) [18]. Notably, the STSS does not separately assess motor and phonic tics, as for example the Yale Global Tic Severity Scale (YGTSS [17]). The total score of the STSS ranges from 0 to 9. These scores are converted into a Global Severity Rating (GSR) ranging from 0 (indicating ‘none’) to 6 (indicating ‘very severe’) as follows: 0 = none, 0–< 1 = very mild, 1–< 2 = mild, 2–< 4 = moderate, 4–< 6 = marked, 6–8 = severe, and > 8–9 = very severe [18]. In addition, we asked for age at tic onset (separately for motor and phonic tics), suppressibility of tics (yes/no), and presence of premonitory urges (PU) (yes/no).

Diagnoses of psychiatric comorbidities such as OCB/OCD, hyperactivity, inattention, rage attacks, anxiety (including different forms of anxiety disorders such as phobias, panic disorders, and general anxiety disorder), depression, sleeping problems, and SIB were made by our principal investigator (KMV) and were based either on patients’ history or—in case of current symptomatology—on DSM/ICD criteria inquired via a semi-structured clinical interview to determine lifetime prevalence for comorbidities. If the patient had either hyperactivity, or inattention, or both, a diagnosis of ADHD was made, thereby forming a single comorbidity. Finally, a comorbidity score was calculated by adding up the total number of comorbidities for each patient, ranging from 0 to 6 (including OCD (but not OCB), ADHD, rage attacks, anxiety, depression, and SIB) as suggested earlier [19]. No additional validated symptom rating scales were used for the assessment of symptom severity of the various clinical symptoms.

The data were gained retrospectively through chart analysis. Data analyses were carried out using the Statistical Package for Social Sciences (V.21.0 for Mac, SPSS Inc.) and Microsoft Excel Mac 2011. *Z* score tests were conducted to look for significant differences in prevalence of various variables in the two groups (TS vs. CMTD). Alpha level was set at 0.05 (two-tailed). For continuous variables, we used *t* tests for independent samples provided by SPSS. Prior to each *t* test, a Levene’s test for equality of variances was applied. The Levene’s test is known to be robust to deviation from normality. Depending on the result of the Levene’s test, the appropriate version of the *t* test was used.

## Results

Of the entire sample of 1018 patients, 771 (77.6%) were males, and 222 (22.4%) were females. While 518 (50.9%) were children (< 18 years old), 500 (49.1%) were adults (≥ 18 years old). Adults were found to be significantly higher on mean tic severity (3.03 vs. 2.58; *p* < 0.001) than children, while not differing on other variables. Nine hundred and seventy-eight (96.1%) patients were diagnosed with TS, whereas the remaining 40 (3.9%) had CMTD. The variation in demographics between these two groups was as follows: among the TS group, 759 (77.6%) were males and 219 (22.4%) were females, whereas among the CMTD group, 28 (70%) were males and 12 (20%) were females; 501 (51.2%) were children and 477 (48.8%) were adults in the TS group, whereas 17 (42.5%) were children and 23 (57.5%) were adults in the CMTD group. Both groups were very comparable in age with the average age for TS being 21.0 years (SD 13.0) and the average age for CMTD being 21.3 years (SD 11.1) (Table 1). Further data based on this sample has been provided elsewhere [20].

**Table 1:** Demographics in the whole sample and by diagnosis

Variables	Whole sample (total *N* = 1018)	TS (*n* = 978)	CMT (*n* = 40)
	*N* (%)/average (SD)	*N* (%)/average (SD)	*N* (%)/average (SD)
Gender			
Male	771 (77.6%)	759 (77.6%)	28 (70%)
Female	222 (22.4%)	219 (22.4%)	12 (20%)
Age group			
Children (< 18 years)	518 (50.9%)	501 (51.2%)	17 (42.5%)
Adults	500 (49.1%)	477 (48.8%)	23 (57.5%)
Diagnosis			
TS	978 (96.1%)		
CMTD	40 (3.9%)		
Average age	21.0 (12.9)	21 (13.0)	21.3 (11.1)

### Age at tic onset in TS vs. CMTD

The mean age at onset of motor tics for those in the TS group was 7.51 years and for those in the CMTD group was 8.18 years. The difference between the means was not found to be significant [*t*(979) = − 1.15; *p* = 0.25].

### Tic severity in TS vs. CMTD

Tic severity expressed by mean GSR according to STSS was 2.83 for the TS group (range 1–6; SD 1.166 [missing data: *n* = 31; 3.16%]) and 2.03 for the CMT group (range 1–5; SD 0.86). A significant difference was found between the two means [*t*(985) = − 4.32; *p* < 0.001]. Table 2 further provides a comprehensive comparison of the varying levels of tic severity between the TS and the CMTD groups.

**Table 2:** Differences in tic severity according to the GSR of the STSS and number of comorbidities for the CMTD and TS groups

Severity	CMTD group number [*n*] (%)	TS group number [*n*] (%)
STSS-GSR		
1 = very mild	12 (30)	103 (10.9)
2 = mild	17 (42.5)	323 (34.1)
3 = medium	9 (22.5)	253 (26.7)
4 = marked	2 (5)	173 (18.3)
5 = severe	0	90 (9.5)
6 = very severe	0	5 (0.5)
Comorbidity score		
0	7 (17.5)	75 (7.7)
1	12 (30)	168 (17.3)
2	6 (15)	202 (20.8)
3	8 (20)	217 (22.3)
4	7 (17.5)	166 (17.1)
5	0 (0)	109 (11.2)
6	0 (0)	36 (3.7)

Comorbidity score ranges from 0 = none to 6

*STSS-GSR* Shapiro Tourette-Syndrome Severity Scale Global Severity Ratings, *CMTD* chronic motor tic disorder, *TS* Tourette syndrome

**Fig. 1:**
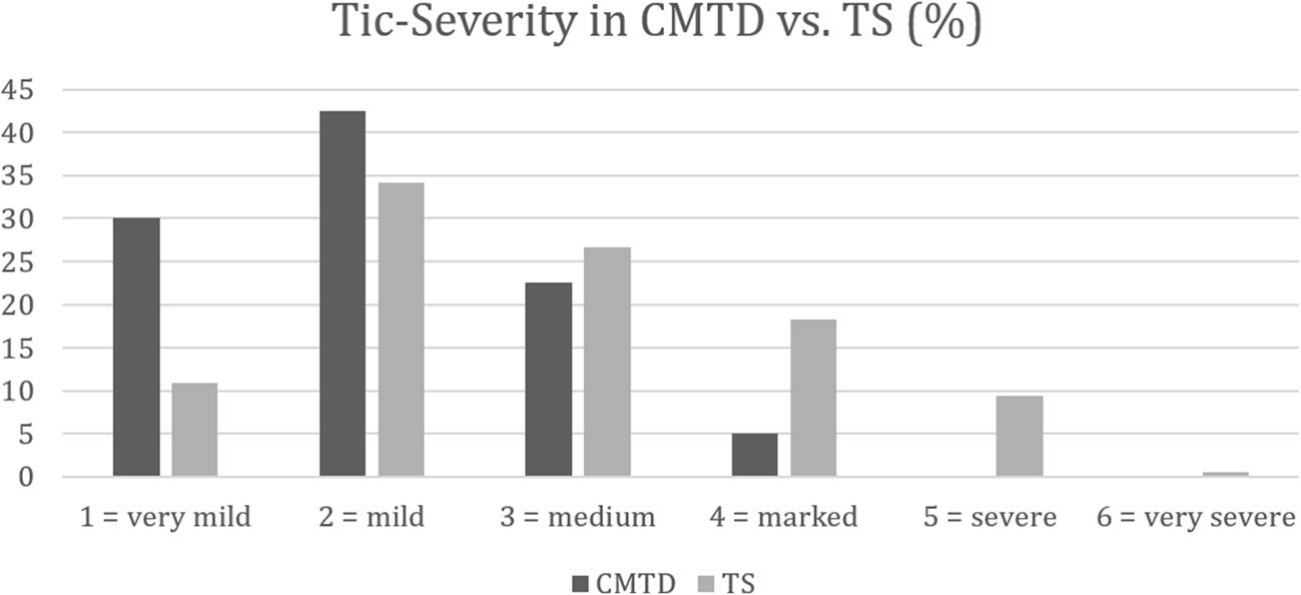
Differences in the distribution of tic severity according to the GSR of the STSS for the CMTD and TS groups. *STSS-GSR* Shapiro Tourette-Syndrome Severity Scale Global Severity Ratings, *CMTD* chronic motor tic disorder, *TS* Tourette syndrome

The difference in tic severity between TS and CMTD remained significant when age was added as a covariate in a linear regression model (beta = 30.4, *p* < 0.001). Within each group, there was no effect of gender [TS: male average SSTS = 2.8 (SD 1.1), females average SSTS = 2.9 (SD 1.3); CMTD: male average SSTS = 2.1 (SD 0.9), female average SSTS = 2.0 (SD 0.7)]. Figure 1 displays the distribution of tic severity for CMTD and TS accross different values of the GSR.

### Simple and complex motor tics in TS vs. CMTD

Nine hundred and seventy-six (99.8%) of the TS group and 40 (100%) of the CMT group had simple motor tics, the difference being insignificant [*z* = − 0.29; *p* = 0.77]. However, results showed a significant difference with respect to complex motor tics, with 546 (55.9%) of the TS group and 11 (27.5%) of the CMT group presenting with them [*z* = 3.527; *p* < 0.01].

### Echopraxia and copropraxia in TS vs. CMTD

Two hundred and thirty-three (23.8%) in the TS group versus 4 (10%) in the CMTD group experienced echopraxia, resulting in a significant difference between the two [*z* = 2.03; *p* < 0.05]. Results showed a significant difference in the prevalence of copropraxia, where 158 (16.2%) versus 0 cases were found in the TS and CMTD groups, respectively [*z* = 2.77; *p* < 0.01].

### Premonitory urge (PU) and tic suppression in TS vs. CMTD

Our results showed that 672 (68.7%) from the TS group and 23 (57.5%) from the CMT group experienced a PU, the difference between them being insignificant [*z* = 1.49; *p* = 0.14].

With respect to tic suppression, 808 (82.6%) and 37 (92.5%) from the TS and CMT groups, respectively, could suppress their tics, thus yielding an insignificant difference between the two proportions [*z* = − 1.63; *p* = 0.10].

### Number and nature of comorbidities in TS vs. CMTD

**Fig. 2:**
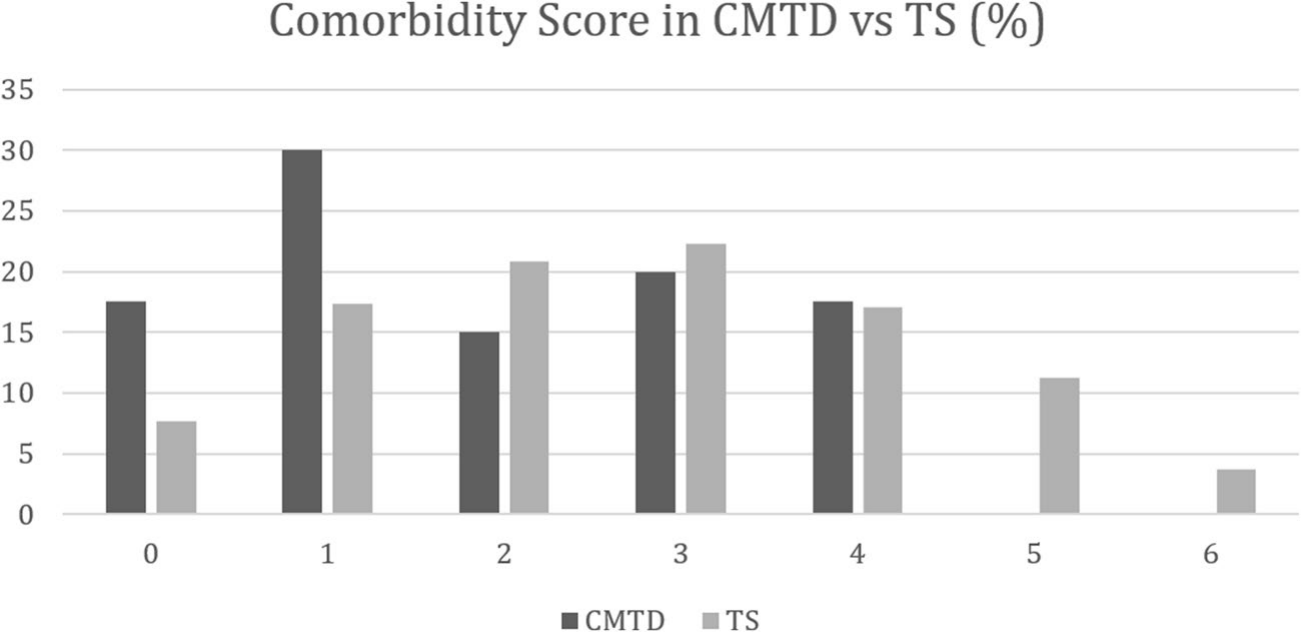
Distribution of the number of comorbidities in the CMTD and TS groups. Comorbidity score ranges from 0 = none to 6; *CMTD* chronic motor tic disorder, *TS* Tourette syndrome

The mean number of comorbidities in the TS group was 2.72 (range 0–6; SD 1.57 [missing data: 5; 0.51%]) and 1.90 (range 0–4; SD 1.39) for the CMTD group. Hence, the TS group had a significantly greater number of comorbidities than the CMTD group [*t*(1011) = − 3.254; *p* < 0.001]. A distribution of the comorbidity score for both groups is displayed on Fig. 2. Table 2 shows the frequency and percentage of patients in each group having different number of comorbidities. Table 3 summarizes the results for various comorbidities. A significant difference (TS > CMTD) was found for the following comorbidities: anxiety, ADHD, SIB, the compulsions of not just right experiences and ordering, and obsessions.

**Table 3:** Difference in prevalence rates of various comorbidities between the CMTD and TS groups

Comorbidity	CMTD group number [*n*] (%)	TS group number [*n*] (%)	Significance
OCD	4 (10)	98 (10)	*Z* = 0; *p* = 1
OCB	20 (50)	612 (62.6)	*Z* = 1.61; *p* = 0.11
Compulsions	24 (60)	707 (72.3)	*Z* = 1.69; *p* < 0.1
Not just right experiences	17 (42.5)	552 (56.4)	*Z* = 1.74; *p* < 0.1
Ordering	4 (10)	234 (23.9)	*Z* = 2.04; *p* < 0.05
Checking	12 (30)	347 (35.5)	*Z* = 0.71; *p* = 0.48
Counting	5 (12.5)	118 (12.1)	*Z* = − 0.08; *p* = 0.94
Washing	2 (5)	84 (8.6)	*Z* = 0.8; *p* = 0.42
Obsessions	9 (22.5)	346 (35.4)	*Z* = 1.68; *p* < 0.1
ADHD	13 (32.5)	448 (45.8)	*Z* = 1.66; *p* < 0.1
Anxiety	6 (15)	314 (32.2)	*Z* = 2.29; *p* < 0.05
Depression	6 (15)	229 (23.4)	*Z* = 1.24; *p* = 0.21
SIB	6 (15)	399 (41)	*Z* = 3.26; *p* < 0.01
Rage attacks	21 (52.5)	564 (57.9)	*Z* = 0.65; *p* = 0.52

*CMTD* chronic motor tic disorder, *TS* Tourette syndrome, *OCD* obsessive–compulsive disorder, *OCB* obsessive–compulsive behavior, *SIB* self-injurious behavior

## Discussion

This paper aims to provide evidence toward the idea that CMTD is a mild form of TS rather than an independent disorder. Hence, the aforementioned factors such as significantly lower mean tic severity, a significantly lower prevalence not only of complex motor tics in general but also of copropraxia, and echopraxia in particular, fewer comorbidities (as indicated by a lower comorbidity score), and markedly lower prevalence of certain comorbidities such as anxiety disorder, ADHD, SIB, certain obsessive–compulsive (OC) symptoms including ordering and not just right experiences, and obsessions in the CMTD group are all in line with the hypothesis. None of the symptoms assessed in this study were found in the CMTD group to be significantly more frequent or more severe compared to the TS group.

Certain results of another recent study that included a large number of patients with both TS and CMTD (*N* = 1018) based on the same sample are highly relevant to the current study [20]. Firstly, results from this recent study [20] as well as other studies [21] showed that comorbidities such as anxiety and depression were more commonly seen along with the more severe forms of TS. In conjunction, current results showed that the prevalence of both these comorbidities as well as certain OC symptoms (not just right feeling, ordering, obsessions, compulsions) was higher in the TS group compared to the CMTD group. Additionally, the difference between the prevalence rates between the TS and CMTD groups was small (though there is a significant difference between the two groups for anxiety, the z-value is relatively small). This further indicates that TS is a more severe form of CMTD and not a separate tic disorder. Secondly, in the large sample [20] we were able to demonstrate that complex tics such as coprophenomena are associated with more severe forms of tic disorders. Accordingly, copropraxia was significantly less common in the CMTD group compared to the TS group. Thirdly, and completely in line with results obtained from another large clinical study [19], in our large sample we found no correlation between tic severity and age at onset of tics, suggesting that age at tic onset is not a predictor for general tic severity later in life. Accordingly, in the current study we also failed to demonstrate a difference between the TS and the CMTD groups with respect to age at tic onset. Notably, predictors that are known to contribute to a poorer quality of life in adulthood are higher tic severity and premonitory urges in childhood as well as a family history of TS prediction [22]. Fourthly, our hypothesis is further supported by the fact that in a recent study, neither significant differences in tic severity between those who could suppress their tics and those who could not, nor a correlation between PU and tic severity was found [20]. Accordingly, in this study, no differences between the TS and CMTD groups could be detected with respect to PU or the ability for tic suppression. These two characteristics of tics also seem to be independent of tic severity. While we assume that TS is in general a more severe form of CMTD, we do not claim that this has always been the case. There are certainly enough individual cases of TS, which are milder than the average CMTD patient; in the same vein, there are certainly enough cases of CMTD which are more severe than that in the typical TS patient. Nevertheless, on average TS patients are more severely affected than CMTD patients.

In a recent Swedish study [23], the validity and inter-rater reliability of tic disorders were investigated using the Swedish National Patient Register. Tic disorder cases showed a very good positive predictive value (PPV) of 0.92 (95% CI 0.82–0.97) when assessed as a whole (ICD-10 code F95). However, there was significantly less agreement regarding the third position of the ICD code (F95.1 = CTD, F95.2 = TS, and F95.9 = unspecified tic disorder) [23]. The authors, therefore, suggested using “simple algorithms” to further increase the confidence in the validity of the diagnostic codes. Thus, in clinical practice it seems to be easy to differentiate between PTD and chronic tic disorder due to the “duration criterion” of tics (less or more than 1 year), but clinicians seem to have difficulties in differentiating between CTD (including CMTD) and TS, and often diagnosing CTD, even when both motor and vocal tics are present [23].

The above-mentioned data point toward an arbitrary distinction made between CMTD and TS. At present, the DSM-5 describes three different primary tic disorders—PTD, CTD and TS—but uses a specifier to differentiate between CMTD and CPTD. However, based on our and others’ findings [14], we propose to revise these categories and instead introduce—comparable to autism spectrum disorder (ASD) in DSM-5—a new term for this group of tic disorders, with PTD falling at one end and severe and complex TS with comorbidities (“TS plus”) being on the other end. CMTD and “pure TS” would lie between the two ends, with the former being on average less severe than the latter. Additionally, a specifier could be provided to differentiate not only between CMTD and—the rare variant—CPTD (as already done in DSM-5), but also between CTD and TS on the one hand and provisional and chronic tic disorders on the other hand. Accordingly, we strongly support the recommendation given by Walkup et al. to not create diagnostic subtypes of TS in the DSM [24]. Finally, one could argue that—following Occam’s razor—the idea that there are several tic disorders is a less parsimonious explanation of the phenomenon than the assumption that there is just one, resulting in the fact that the “more complicated assumption” needs clear scientific proof as opposed to the simpler one. Thus, without adequate proof for the more complicated assumption, we should assume there is just one diagnosis to be given. Further evidence for CMTD and TS being a unified condition comes from a very recent genome-wide association study meta-analysis in 4819 TS cases and 9488 controls from a population-based Icelandic sample. The analysis demonstrated that TS and other tic disorders share the same polygenic risk scores, which supports the idea of a unified condition [25].

We, therefore, suggest the introduction of a revised diagnosis of “tic spectrum disorder” (TSD) as a more accurate, medically and scientifically useful way of diagnosing individuals with primary tic disorders (including PTD, CTD, and TS). Since the term “TS” has a long tradition, and most doctors, patients, and other lay persons associate a “chronic combined phonic and motor tic disorder” with this term, we suggest continued use of this term—instead of complete deletion—no longer as a separate diagnosis, but as a variation of a primary tic disorder that can be defined by using specifiers. In addition to this scientific argument in favor of the term “tic spectrum disorder”, we should take into consideration an aspect, which is important from the patient’s perspective. Mainly due to the frequent presentation of severe and coprolalic TS in the media, the terms “Tourette” and “Tourette syndrome”, respectively, carry a certain amount of stigma. Understandably, more and more patients prefer to avoid this term. Therefore, the introduction of the term “tic spectrum disorder” would make it possible for both, treating physicians and their patients, to avoid the term “TS” without evading the correct diagnosis.

One of the limitations of this study is that the DSM went through a few changes in diagnostic criteria over the time period during which the data used for this study were collected. One of the most influential changes was the dismissal of the “impairment criterion” in the DSM starting from DSM-IV-TR. However, no changes were made with respect to the differences between TS and CMTD. Also, since the ICD did not include the “impairment criterion” during this time, the change in the DSM was not reflected in our diagnostic assessments. Although this is the largest study of this kind, the number of patients with CMTD was still relatively small. Based on the years of extensive clinical experience as well as the data presented, we believe that the number of patients with CMTD presenting in a specialized Tourette outpatient clinic is relatively small, because in most cases CMTD does not result in a significant impairment in patients’ quality of life due to its general mild symptom presentation [22, 26]. We believe that even a trend such as the one observed in the current results (providing a *p* value of less than 0.10 and not necessarily *p* < 0.05) could be an interesting finding. Finally, we did not use standardized assessments, but used a semi-structured interview for the diagnoses of comorbidities. However, our main findings are based on tic-related aspects and not on comorbid psychiatric symptoms. Unfortunately, the clinical data on CMTD is still extremely limited. Although most clinicians feel that CMTD is a mild form of TS, we can say that to the best of our knowledge there is no robust literature present suggesting the same. Thus, this paper is the first of its kind and attempts to fill the gap in research literature by providing a comprehensive comparison between the two phenomena with respect to various clinical aspects.

In conclusion, our data strongly support the idea of a spectrum with CMTD being—on average—only a less severe presentation of TS with respect to all clinical symptoms including number, frequency, complexity, and severity of motor and vocal tics, as well as number and severity of comorbidities, but without any difference in age at tic onset, PU, and tic suppressibility. We therefore suggest that the new term “tic spectrum disorder” be introduced to further differentiate between different variants depending on the duration and kind of tics.

## Electronic supplementary material

Below is the link to the electronic supplementary material.

Supplementary material 1 (PDF 235 kb)
