# Supplementary material for: Tic disorders revisited: introduction of the term “tic spectrum disorders”
Source: Eur Child Adolesc Psychiatry. 2019 Jan 19;28(8):1129–35. doi: 10.1007/s00787-018-01272-7 (PMC6675752; doi:10.1007/s00787-018-01272-7)
Supplement: Supplementary file 1 — Supplementary material 1 (PDF 235 kb) [file 787_2018_1272_MOESM1_ESM.pdf]

frontiers in Neuroscience

ORIGINAL RESEARCH
published: 12 September 2016
doi: 10.3389/fnins.2016.00415

New Insights into Clinical Characteristics of Gilles de la Tourette Syndrome: Findings in 1032 Patients from a Single German Center

Tanvi Sambrani[1,2]*, *Ewgeni Jakubovski*[2] *and Kirsten R. Müller-Vahl*[2]

[1] *Department of Education, Monash University, Melbourne, VIC, Australia,* [2] *Clinic of Psychiatry, Social Psychiatry, and Psychotherapy, Hannover Medical School, Hannover, Germany*

OPEN ACCESS

Edited by:
Ashok Mysore,
St. John's Medical College Hospital, India

Reviewed by:
Rajshekhar Bipeta,
Gandhi Medical College and Hospital, India
Kerstin Jessica Plessen,
University of Copenhagen, Denmark
Janardhan Y. C. Reddy,
National Institute of Mental Health and Neurosciences, India

***Correspondence:**
Tanvi Sambrani
tsam14@student.monash.edu

Specialty section:
This article was submitted to Child and Adolescent Psychiatry, a section of the journal Frontiers in Neuroscience

Received: *23 March 2016*
Accepted: *26 August 2016*
Published: *12 September 2016*

Citation:
Sambrani T, Jakubovski E and Müller-Vahl KR (2016) New Insights into Clinical Characteristics of Gilles de la Tourette Syndrome: Findings in 1032 Patients from a Single German Center. Front. Neurosci. 10:415. doi: 10.3389/fnins.2016.00415

Background: Gilles de la Tourette syndrome (TS) is a complex neuropsychiatric disorder defined by the presence of motor and phonic tics, but often associated with psychiatric comorbidities. The main objective of this study was to explore the clinical presentation and comorbidities of TS.

Method: We analyzed clinical data obtained from a large sample ($n = 1032$; 529 children and 503 adults) of patients with tic disorders from one single German TS center assessed by one investigator. Data was collected with the help of an expert-reviewed semi-structured interview, designed to assess tic severity and certain comorbidities. Group comparisons were carried out via independent sample t-tests and chi-square tests.

Results: The main findings of the study are: (1) tic severity is associated with the presence of premonitory urges (PU), copro-, echo-, and paliphenomena and the number of comorbidities, but not age at tic onset; it is higher in patients with comorbid obsessive-compulsive disorder (OCD) than in patients with comorbid attention deficit/hyperactivity disorder (ADHD). (2) PU were found to be highly associated with "not just right experiences" and to emerge much earlier than previously thought alongside with the ability to suppress tics (PU in >60% and suppressibility in >75% at age 8–10 years). (3) Self-injurious behavior (SIB) is highly associated with complex motor tics and coprophenomena, but not with OCD/obsessive-compulsive behavior (OCB). While comorbid ADHD is associated with a lower ability to suppress tics, comorbid depression is associated with sleeping problems.

Discussion: Our results demonstrate that tic severity is not influenced by age at onset. From our data, it is suggested that PU represent a specific type of "not just right experience" that is not a prerequisite for tic suppression. Comorbid ADHD reduces patients' ability of successful tic suppression. Our data suggest that SIB belongs to the

coprophenomena spectrum and hence should be conceptualized as a complex tic rather than a compulsion. Finally, this study strongly supports the hypothesis that TS+OCD is a more severe form of TS and that comorbid OCD/OCB, depression, and anxiety belong to the TS spectrum, while ADHD should be better conceptualized as a separate problem.

Keywords: Tourette syndrome, tics, comorbidities, ADHD, OCD, premonitory urges, self-injurious behavior, depression

INTRODUCTION

A part of the broad spectrum of tic disorders—including provisional tic disorder and persistent (chronic) tic disorder—Gilles de la Tourette syndrome (TS) is a neuropsychiatric disorder with an onset in childhood. This developmental disorder is characterized by the presence of multiple motor tics and at least one phonic tic for a minimum period of 1 year, beginning before 18 years of age and occurring in bouts (American Psychiatric Association, 2013). Typically, tics begin to appear between 5–7 years of age, follow a waxing and waning course, reach their peak severity between 10 and 12 years of age, and decline drastically or even vanish completely by the end of adolescence (Leckman et al., 1998). Not only is TS found among people in all countries, cultures, and ethnic groups, but its main characteristics are also mostly similar across such different populations (Tanner and Goldman, 1997), with the estimated prevalence ranging between 1% (Robertson et al., 2009) and 5.26% (Cubo et al., 2011), the latter relatively high percentage conveying that TS in the general community is milder and more ubiquitous than its prevalence estimates based on its occurrence in clinical settings (Robertson, 2000). Relevant literature also points toward a clear sex difference in prevalence of TS, with it being approximately four times more common in males than in females (Robertson, 1994; Tanner and Goldman, 1997; Freeman et al., 2000).

The vast majority of individuals with TS report a certain discomfort or feeling of pressure before a tic occurs—either focal and specific to a particular anatomical location or more generalized—called "premonitory urge" (PU), that tends to intensify before the tic occurs and is usually mitigated temporarily once the individual performs the tic (Leckman et al., 1993).

Particularly in more severely affected patients, simple motor and phonic tics are often accompanied by different forms of complex tics including coprophenomena—such as making socially inappropriate gestures (copropraxia) or verbal expressions (coprolalia)—in which symptoms tend to manifest during the preadolescent period when tics are at their most severe (Freeman et al., 2009), echophenomena such as imitating gestures (echopraxia) and words or phrases (echolalia), and paliphenomena such as phonic blocking and repetition (palilalia). Additionally, TS is considerably comorbid with attention deficit/hyperactivity disorder (ADHD), obsessive-compulsive disorder (OCD) or (subclinical) obsessive-compulsive behavior (OCB), anxiety disorders, depression, self-injurious behavior (SIB), rage attacks, and to a lesser extent with learning disorders, personality disorders, and other behavioral disorders (Robertson, 2000). Freeman et al. (2000) found that at all ages, a mere 12% of the clinical population with TS have no reported comorbidity.

Robertson and Baron-Cohen (1998) have provided a relatively clear clinical division of TS: (i) "pure TS," including almost only motor and phonic tics; (ii) "full blown TS," consisting of related echo-, copro-, and paliphenomena; and (iii) "TS plus" (first coined by Packer, 1997), which includes cases of those who also have SIB, ADHD, severe OCB, or OCD, as well as other severe psychopathologies such as depression, anxiety, and antisocial behaviors. Another classification was proposed by Robertson and Cavanna (2007), in which "cluster 1" consisted of only simple tics, "cluster 2" involved tics, ADHD, and aggression, whilst "cluster 3" included tics, and affective disorders such as OCD/OCB, depression, and anxiety. The second classification is in line with research indicating that depression, anxiety, OCD, and OCB are all a part of the TS spectrum, whereas ADHD is somewhat separate (Lebowitz et al., 2012; Hirschtritt et al., 2015; Trillini and Müller-Vahl, 2015).

In their multisite study, Freeman et al. (2000) analyzed data obtained from 3500 individuals diagnosed with TS obtained from 64 different centers across 22 countries, and established several findings. They reported that the mean age at onset of tics was 6.4 years. The most common reported comorbidity was ADHD at 60% followed by history of sleeping problems (37%) and OCB (32%). In terms of gender differences, they confirmed not only the well-known male:female ratio (4.3:1), but also found that males are more likely to have comorbid disorders such as conduct disorder (CD)/oppositional defiant disorder (ODD), ADHD, pervasive developmental disorders, anger control problems, specific learning disability, stuttering, social skills problems, and pre-/perinatal-problems than females, whereas females showed significantly higher rates for SIB and trichotillomania. Males were also found to have a significantly higher comorbidity score (2.06) than females (1.83). Symptoms such as sleeping problems, coprolalia, SIB, and anger control problems were found to be positively associated with the comorbidity score. With respect to associated psychopathology, their results also showed that sleeping problems were twice as likely to occur in the comorbid group as opposed to the "TS only" group (without comorbidities).

Through this study, we aim to extend our knowledge of possible differences in the clinical presentation and nature of TS-related phenomena/various tic symptoms based on gender, age groups, and single comorbidity subgroups of TS, and thereby achieve a better understanding of the factors producing variations in order to engage in better subtyping of TS. Therefore, we used a large database—consisting purely of data gathered during the first evaluation of every patient at the TS center—as

a suitable starting point for studying a large sample ($n = 1032$) from a single research site, thereby controlling for inter-site variance. We also posit a more specific hypothesis that whilst comorbidities such as OCD, anxiety, depression, and SIB are more related to the TS spectrum, comorbidities like ADHD and rage attacks tend to be slightly independent of it, as has been suggested earlier (Lebowitz et al., 2012; Hirschtritt et al., 2015; Trillini and Müller-Vahl, 2015).

METHODS

This study bases on a large data set obtained from only one single large German TS center at the Clinic of Psychiatry, Socialpsychiatry, and Psychotherapy at the Hannover Medical School (MHH). It is the largest TS center in all of Germany with an outpatient clinic which treats both adults and children. The waiting time for an appointment ranges between 2 and 3 months. The administration of this institution allows patients to visit the outpatient clinic without the need for a referral by another medical professional. The medical costs incurred by the patients' families are covered by health insurance. Thus, patients from all over Germany—and in some cases even from abroad—present at this TS center. All patients included in this study had not only been personally seen by one of the authors (KMV), but their medical history was also personally looked into by her in each case. KMV is both a neurologist and an adult psychiatrist and a well-experienced specialist for tic disorders.

Clinical data was elicited over a period of nearly 20 years (1995–2013) from 1032 patients consisting of 529 children and 503 adults (median age = 17; $SD = 12.91$) with various tic disorders. The diagnoses of different form of tic disorders were made according to DSM criteria valid at that time (DSM-III-R–DSM-IV-TR). All subjects were prospectively interviewed using a clinician-reviewed semi-structured interview assessing several different aspects including tics and comorbidities. This schedule closely resembles the National Hospital Interview Schedule, developed by Robertson and Eapen (1996), and its credibility was established based on the expertise of one of the authors (KMV). Lifetime data for 9 motor and phonic tic symptoms were obtained: simple motor (MT) and phonic tics (PT), complex MT and PT (including all different forms of complex tics), and specifically coprolalia, copropraxia, echolalia, echopraxia, and palilalia (each symptom was scored as either present or absent). In addition, we asked for age at tic onset (separately for MT and PT), suppressibility of tics (yes/no), and presence of PU (yes/no), including the nature/localization of the PU (local, diffused, or uncertain).

Current tic severity was assessed on the day of diagnosis at MHH using the Shapiro Tourette-Syndrome Severity Scale (STSS), which contains five variables with matching rating scales as follows: (1) tics noticeable to others (0–3), (2) tics elicit comments or curiosity (0–1), (3) patient considered odd or bizarre (0–2), (4) tics interfere with functioning (0–2), (5) incapacitated, homebound or hospitalized (0–1). The total score ranges from 0 to 9 with the following interpretation: 0 = none, $0 - <1$ = very mild, $1 - <2$ = mild, $2 - <4$ = moderate, $4 - <6$ = marked, $6 - 8$ = severe, and $>8 - 9$ = very severe (Shapiro et al., 1988). The corresponding Global Severity Ratings (GSR) range from 0 (indicating "none") to 6 (indicating "very severe").

Lifetime prevalence for the following comorbidities was evaluated based on the semi-structured clinical interview: hyperactivity, inattention, rage attacks, anxiety (including different forms of anxiety disorders including phobias, panic disorders, and general anxiety disorder), depression, OCD, SIB, and sleeping problems. ADHD was diagnosed for each participant based on the presence of either hyperactivity or inattention, and hence these two are considered one comorbidity. OCB, being a mild form of OCD, was assessed, but not considered as a comorbidity. Specific obsessive-compulsive (OC) symptoms such as obsessions and compulsions—particularly those of counting, checking, ordering, washing, and "not just right experiences"—were also assessed. For the assessment of comorbidities, no validated rating scales were used. Diagnoses of psychiatric comorbidities were based either on patients' history or—in case of current symptomatology—on DSM criteria. A comorbidity score was calculated by adding up the total number of comorbidities for each patient as suggested earlier (Freeman et al., 2000), ranging from 0 to 6 (including OCD, ADHD, rage attacks, anxiety, depression, and SIB).

Several group comparisons were undertaken. Patients diagnosed with TS according to DSM ("TS group") were categorized as "TS only"—consisting of those individuals with no comorbidities (as defined above)—and "TS + comorbidities" (equivalent of the "TS plus" sub-group coined by Packer, 1997)—comprised those TS patients with ≥1 comorbidity (including OCD, ADHD, rage attacks, anxiety, depression, and SIB). In addition, we conducted two further subgroup comparisons: (i) TS+OCD (excluding ADHD but including other comorbidities) vs. TS+ADHD (excluding OCD/OCB but including other comorbidities) in order to further investigate the hypothesis that OCD, but not ADHD, is part of the TS spectrum; and (ii) TS+OCD/OCB/anxiety/depression/SIB vs. TS+ADHD+rage attacks in order to verify the validity of the classification of TS suggested by Robertson and Cavanna (2007).

All data analyses were carried out using the Statistical Package for Social Sciences (V.21.0 for Mac, SPSS Inc.) and Microsoft Excel Mac 2011. Z-score tests of proportion were carried out to measure gender differences for various clinical features of TS and associated disorders. Pearson's Chi-square tests were conducted to test for associations between categorical variables measuring the presence of several comorbidities. ANOVA and t-tests were conducted to test for gender differences and differences in tic severity in continuous variables. Alpha value was set at 0.05 (two-tailed). Due to a few issues related to data collection, some of the fields in the database were left blank, thereby resulting in an incomplete database and consequently certain missing values, the absolute numbers and percentages of which have been mentioned at the appropriate parts of the results section.

RESULTS

Of the entire sample of 1032 patients, 529 (51.3%) were children (<18 years of age) whilst 503 (48.7%) were adults (≥18 years of age). Nine hundred and seventy-eight patients

had TS, 40 patients were diagnosed with chronic motor tics (CMT), and the remaining 14 had other tic disorders, such as transient (provisional) or chronic (persistent) vocal or other tic disorders. Unless otherwise specified, all the results pertain to the entire sample. Further, details for the CMT group will be provided elsewhere (Müller-Vahl et al., in preparation). Depending on the presence of comorbidities we found: "TS only" ($n = 75$; 7.2%), "TS + comorbidities" ($n = 898$; 87.0%), TS+OCD ($n = 45$; 4.4%), TS+ADHD ($n = 102$; 9.9%), TS+OCD/OCB/anxiety/depression/SIB ($n = 209$; 20.3%), and TS+ADHD+rage attacks ($n = 56$; 5.4%).

Tics: Age at Onset and Tic Severity

The mean age at time of assessment was 20.9 years (range 4–72; *SD*: 12.91). The mean age at onset of tics was 6.97 years (range, 0–21; *SD*: 3.17). Only one of the 978 participants claimed that his tics started at the age of 21 years, although all other respective DSM criteria were met. The mean age at onset for MT was 7.51 years (range, 0–53; *SD*: 3.95) whereas that for PT was 9.76 years (range, 0–48; *SD*: 5.58). The mean age at diagnosis was 18.9 years (range, 4–72; *SD*: 12.72). Mean tic severity ratings (GSR) according to STSS were 2.79 when considering the entire sample (range, 1–6; *SD*: 1.17) and 2.83 within the TS group (range, 1–6; *SD*: 1.17). **Table 1** shows tic severity across the entire sample demonstrating that even in a tertiary referral center like ours, tic severity is very mild to moderate in almost three-quarter of patients. No significant relationship was found between age at onset of tics and tic severity as measured by the STSS [$F_{(5)} = 0.817$; n.s.]. **Table 2** shows percentages of the total sample developing tics at various age ranges.

TABLE 1 | Tic severity according to STSS-GSR (range, 1–6) across the sample (*n* = 1032).

STSS-GSR	Number (%)
1 = very mild	120 (12.0)
2 = mild	343 (34.3)
3 = medium	265 (26.5)
4 = marked	176 (17.6)
5 = severe	90 (9.0)
6 = very severe	5 (0.5)

STSS-GSR, Global Severity Rating of the Shapiro Tourette-Syndrome Severity Scale.

TABLE 2 | Age range at tic onset.

Age range [years]	Tic onset *N* (%)
<6	352 (34.1)
6–10	543 (52.6)
11–15	119 (11.6)
16–18	17 (1.6)
>18	1 (0.1)

N = number of cases.

Considering the three age groups (based on current age of the patient) according to age dependency of tic severity provided by Bloch et al. (2006), we found a significant positive association between mean tic severity and age groups [$F_{(2)} = 31.658$; $p < 0.001$]: age <10 years ($n = 184$): mean STSS-GSR = 2.33, age 10–12 years ($n = 198$): mean STSS-GSR = 2.52, and age >12 years ($n = 650$): mean STSS-GSR = 3.00. For the purpose of finding the age group where tics reach their worst severity, in addition, for the following age groups mean STSS-GSR were calculated and again demonstrated a significant positive relationship between STSS-GTS and age groups [$F_{(5)} = 12.688$; $p < 0.001$]: <6 years ($n = 13$): mean STSS-GSR = 2.31, 6–10 years ($n = 251$): mean STSS-GSR = 2.38, 11–15 years ($n = 205$): mean STSS-GSR = 2.66, 16–20 years ($n = 141$): mean STSS-GSR = 2.99, 21–25 years ($n = 100$): mean STSS-GSR = 3.02, >25 years ($n = 322$): mean STSS-GSR = 3.06.

In addition, mean tic severity (STSS-GSR) was found to be highly associated with the comorbidity score [$F_{(6)} = 19.945$; $p < 0.001$; **Table 3**]. Mean STSS-GTS was significantly lower in the "TS only" group (2.20) compared to the "TS + comorbidity" group [2.88, [$t_{(945)} = -4.937$; $p < 0.001$]]. Further, details regarding tic severity depending on the presence of one or more comorbidities are given in **Table 4**.

Coprophenomena, Echophenomena, Palilalia

With regards to specific complex tics ("full blown TS"), 290 patients (28.1%) reported coprophenomena, of which 247 (24%) reported coprolalia and 160 (15.5%) copropraxia, 290 patients (28.1%) claimed to have echolalia and 238 (23.1%) echopraxia, and 339 patients (33%) reported palilalia. Results showing associations between presence of various complex tics and tic severity are shown in **Table 5**.

In addition, we found a significant association between coprophenomena and comorbidity score ($X^2 = 126.823$;

TABLE 3 | Association between number of comorbidities (=comorbidity score) and mean tic severity*.

Number of comorbidities	*N*	Mean tic severity	*SD*	Std. Error	95% Confidence Interval for Mean	
					Lower bound	Upper bound
0	82	2.11	0.817	0.09	1.93	2.29
1	179	2.38	1.006	0.075	2.23	2.53
2	206	2.71	1.083	0.075	2.56	2.86
3	217	2.82	1.147	0.078	2.67	2.97
4	170	3.02	1.186	0.091	2.84	3.20
5	107	3.38	1.264	0.122	3.14	3.63
6	36	3.69	1.091	0.182	3.33	4.06

**comorbidity score including OCD, anxiety, depression, SIB, rage attacks, and ADHD, mean tic severity (according to SPSS-GSR; missing data: n = 35).*
OCD, obsessive-compulsive disorder; OCB, obsessive-compulsive behavior; ADHD, attention-deficit hyperactivity disorder; SIB, self-injurious behavior; STSS-GSR, Global Severity Rating of the Shapiro Tourette-Syndrome Severity Scale; N, number of cases; SD, standard deviation; Std., Standard.

TABLE 4 | Tic severity depending on the presence (±) of different comorbidities*.

Comorbidity		N	Mean tic severity	SD	Std. Error Mean	t-value	Significance
OCD†	+	97	3.15	1.143	0.038	3.266	$p < 0.001$
	−	901	2.74	1.307	0.133		
Anxiety	+	314	3.05	1.106	0.042	4.88	$p < 0.001$
	−	684	2.67	1.250	0.071		
Depression	+	228	3.15	1.130	0.041	5.453	$p < 0.001$
	−	771	2.68	1.218	0.081		
SIB	+	392	3.19	1.039	0.042	9.201	$p < 0.001$
	−	604	2.52	1.235	0.062		
Rage attacks	+	577	2.96	1.081	0.053	5.642	$p < 0.001$
	−	420	2.54	1.198	0.050		
ADHD†	+	449	2.92	1.174	0.050	3.256	$p < 0.001$
	−	550	2.68	1.146	0.054		
TS only		75	2.20	0.870	0.100	−4.937	$p < 0.001$
TS + comorbidities		872	2.88	1.172	0.04		
TS+OCD		45	3.07	1.388	0.209	2.434	$p < 0.05$
TS+ADHD		102	2.57	0.956	0.099		
TS+OCD/OCB/Anxiety/Depression/SIB		205	2.66	0.908	0.125	1.424	n.s.
TS+ADHD+rage attacks		53	2.42	1.155	0.081		

*comorbidities including OCD, anxiety, depression, SIB, rage attacks, and ADHD, mean tic severity according to SPSS-GSR (missing data: n = 35).
†groups include patients having other miscellaneous comorbidities and are therefore larger in number than the "TS+OCD" and "TS+ADHD" groups, which indicate a purer form of the stated comobidity.
OCD, obsessive-compulsive disorder; OCB, obsessive-compulsive behavior; ADHD, attention-deficit hyperactivity disorder; SIB, self-injurious behavior; STSS-GSR, Global Severity Rating of the Shapiro Tourette-Syndrome Severity Scale; N, number of cases; SD, standard deviation; Std., Standard.

TABLE 5 | Tic severity* depending on presence (±) of certain complex tics.

Complex tic		N	Mean STSS-GSR	SD	Std. error	t-value	Significance
Coprophenomena	+	278	3.36	1.255	0.075	−10.093	$p < 0.001$
	−	721	2.57	1.052	0.039		
Coprolalia	+	239	3.46	1.262	0.082	−10.742	$p < 0.001$
	−	757	2.58	1.050	0.038		
Copropraxia	+	154	3.49	1.280	0.103	−8.473	$p < 0.001$
	−	843	2.66	1.096	0.038		
Echolalia	+	280	3.21	1.220	0.073	−7.438	$p < 0.001$
	−	717	2.62	1.100	0.041		
Echopraxia	+	227	3.19	1.292	0.086	−6.101	$p < 0.001$
	−	770	2.67	1.098	0.040		
Palilalia	+	325	3.22	1.175	0.065	−8.406	$p < 0.001$
	−	670	2.58	1.104	0.043		

*according to STSS-GSR.
STSS-GSR, Global Severity Rating of the Shapiro Tourette-Syndrome Severity Scale; N, number of cases; SD, standard deviation; Std., Standard.

$p < 0.001$), echophenomena and comorbidity score ($X^2 = 76.14$; $p < 0.001$), and paliphenomena and comorbidity score ($X^2 = 87.38$; $p < 0.001$). Prevalence rates of coprophenomena demonstrated a significant association with age ($X^2 = 23.227$; $p < 0.001$): <10 years = 36 (19.6%), 10–12 years = 46 (23.2%), and >12 years = 208 (32%). There were also significant associations between age group and other complex tics such as echolalia ($X^2 = 7.736$; $p < 0.05$), echopraxia ($X^2 = 24.737$; $p < 0.001$), and palilalia ($X^2 = 7.111$; $p < 0.05$). In terms of individual comorbidities, coprophenomena were most highly associated with SIB ($X^2 = 60.302$; $p < 0.001$; with both coprolalia and copropraxia having similarly significant associations), followed by depression ($X^2 = 34.484$; $p < 0.001$), rage attacks ($X^2 = 33.800$; $p < 0.001$); ADHD ($X^2 = 30,856$; $p < 0.001$); anxiety ($X^2 = 27.122$; $p < 0.001$), OCD ($X^2 = 17.341$; $p < 0.001$), and lastly OCB ($X^2 = 13.551$; $p < 0.001$).

Premonitory Urges (PU) and Tic Suppressibility

Of the total sample of 1032 participants, 291 (29.4%) individuals did not report a PU [missing data, $n = 41$ (3.97%)]. Amongst the 700 (67.82%) patients who did report the experience of a PU, 446 (46%) participants experienced a localized PU, 113 (11.6%) a diffused PU, and for the remaining 141 (13.7%), although the PU was present, its precise nature was uncertain. With regards to tic suppression, 853 (85.4%) participants mentioned that they were able to suppress their tics whereas 146 (14.6%) claimed that they were unable to do so (missing data, $n = 33$).

In order to investigate age dependencies for both PU and suppressibility, we used different age ranges: on the one hand we used ranges based on the natural course of tics with the worst period between ages 10 and 12 (Bloch et al., 2006)—<10 years, 10–12 years, >12 years (**Table 6**)—and on the other hand age groups as suggested by Banaschewski et al. (2003): 8–10, 11–14,

15–19 years. In addition to the latter age ranges, we looked at PU and suppressibility in very young children (age <8 years) and adults (age >19 years) to further investigate age dependency and possible habituation (**Table 7**). Both classifications demonstrated clear age dependencies for PU as well as tic suppression. Patients who reported a PU suffered from significantly more severe tics compared to those without PU [mean STSS-GSR = 2.87 vs. 2.62, ($t = -3.164$; $p < 0.005$)]. In contrast, tic severity was not different in patients who were able to suppress their tics compared to those who were unable to do so [mean STSS-GSR = 2.83 vs. 2.63, ($t = -1.830$; n.s.)]. A significant positive association was found between PU and the ability to suppress tics ($X^2 = 96.691$; $p < 0.001$).

While no significant association was found between PU and OCD ($X^2 = 3.085$; n.s.), we found a highly significant ($X^2 = 15.379$; $p < 0.001$) positive association between PU and OCB. In particular, there was a strong direct association between PU and "not just right experiences" ($X^2 = 20.871$; $p < 0.001$). Certain other such significant positive associations were also found between PU and obsessions ($X^2 = 11.218$; $p < 0.01$), compulsions in general ($X^2 = 26.769$; $p < 0.01$), and various specific OC symptoms including counting ($X^2 = 15.571$; $p < 0.01$), checking ($X^2 = 18.897$; $p < 0.01$), ordering ($X^2 = 13.979$; $p < 0.01$), but not washing ($X^2 = 0.854$; n.s.). Tic suppression was found to have a significant and direct association with inattention ($X^2 = 6.056$; $p < 0.05$), rage attacks ($X^2 = 5.062$; $p < 0.05$), and hyperactivity ($X^2 = 4.838$; $p < 0.05$).

TABLE 6 | PU, tic suppression, comorbidity score, and tic severity* based on age ranges suggested by Bloch et al. (2006).

Age group (years; *N*)	PU reported (%)	Ability to suppress tics (%)	Mean comorbidity score (entire age group)	Mean tic severity (entire age group)
<10 (181)	46.7	65.5	2.35	2.33
10–12 (195)	61.3	78.8	2.21	2.52
>12 (623)	79.7	92.6	2.91	3

*according to STSS-GSR

PU, premonitory urge; STSS-GSR, Global Severity Rating of the Shapiro Tourette-Syndrome Severity Scale; N, numbers.

Comorbidities

Comorbidity Score

Across the entire sample, following were the percentages of different comorbidity scores: 84 patients (8.2%) had no comorbidity ("TS only"), 186 (18.1%) had one, 211 (20.5%) had two, 226 (22%) had three, 175 (17%) had four, 109 (10.6%) had five, and 36 (3.5%) had all six comorbidities as defined above (OCD, anxiety, depression, SIB, rage attacks, and ADHD; missing data: $n = 5$; see also **Table 3**). The mean comorbidity score was 2.67 (range, 0–6; $SD = 1.57$). A significant positive relationship was found between age groups and comorbidity score [$F_{(2)} = 20.579$; $p < 0.001$; **Tables 6, 7**]. Only 75 (7.67%) of the individuals from the "TS group" presented with "TS only." **Table 4** shows that in all those with a comorbidity score $\geq$1 (indicating the presence of any of the six comorbidities), the mean tic severity tended to be significantly higher than in those with no comorbidity. We also ran a test to compare the presence of comorbidities in patients based on the following age groups: <25, ≥25 and <50, and ≥50 years at the time of assessment. This was done to check for recall issues in describing comorbidities before and after a certain age. However, no significant differences were found between the various age groups.

OCD/OCB

Whilst only 103 (10%) patients of the sample had been clinically diagnosed with OCD according to DSM, 637 (61.8%) suffered from mild to moderate OCB without fulfilling diagnostic criteria for OCD. **Table 8** shows prevalence rates for different forms of OC symptoms amongst those with OCD and OCB. The most often reported OC symptom was a "not just right experience" [in 575 (55.9%) patients across the entire sample].

ADHD, Rage Attacks

Hyperactivity was reported by 291 (28.4%) participants, inattention by 405 (39.4%), and the diagnosis of ADHD according to DSM was made in 463 (44.9%) individuals. As expected, ADHD was significantly associated with hyperactivity ($X^2 = 499.818$; $p < 0.001$), inattention ($X^2 = 816.434$; $p < 0.001$), and rage attacks ($X^2 = 67.331$; $p < 0.01$). However, the association with rage attacks was comparatively much weaker than that with the other two variables. Of the 594 patients (57.8%) who were diagnosed with rage attacks, 261 (43.94%) did

TABLE 7 | PU, tic suppression, comorbidity score, and tic severity* compared to results provided by Banaschewski et al. (2003).

Age group years (*N*)	Presence of premonitory feeling		Ability to suppress tics		Mean comorbidity score (entire age group)	Mean tic severity (entire age group)
	Banaschewski et al. (%)	Current study (%)	Banaschewski et al. (%)	Current study (%)		
<8 (80)	–	34.8	–	56.5	2.23	2.34
8–10 (180)	34	61.8	48	75.1	2.36	2.39
11–14 (174)	56	61.8	66	82.8	2.35	2.66
15–19 (136)	68	76.6	79	90.6	2.62	2.92
>19 (429)	–	81.3	–	93.4	3.03	3.05

*according to STSS-GSR, comorbidity score including OCD, anxiety, depression, SIB, rage attacks, and ADHD.

PU, premonitory urge; STSS-GSR, Global Severity Rating of the Shapiro Tourette-Syndrome Severity Scale.

TABLE 8 | Prevalence rates of different forms of OC symptoms in TS patients with comorbid OCD compared to those with comorbid OCB.

OC Symptom	OCD (*N*; %)	OCB (*N*; %)	*Z*-score	Significance
Compulsions	101 (98.1)	603 (94.9)	1.41	n.s.
-Not just right experiences	87 (85.3)	467 (73.3)	2.38	n.s.
-Checking	77 (76.2)	279 (43.9)	5.02	$p < 0.001$
-Ordering	57 (55.9)	181 (28.4)	3.80	$p < 0.001$
-Washing	35 (34.7)	50 (7.8)	3.12	$p < 0.01$
-Counting	28 (27.7)	96 (15.1)	1.53	n.s.
Obsessions	92 (91.1)	262 (41.3)	8.25	$p < 0.001$

OC, obsessive-compulsive; OCD, obsessive-compulsive disorder; OCB, obsessive-compulsive behavior; TS, Tourette Syndrome; N, numbers; n.s., not significant.

not suffer from either hyperactivity or inattention, thus giving rise to the new and significant observation of the prevalence of rage attacks in Tourette patients, often even in the absence of ADHD.

Sleeping Problems

Sleeping problems (lifetime prevalence) were reported by 273 (26.7%) patients in the sample. Sleeping problems were found to have strong direct associations with depression ($X^2 = 24.548$; $p < 0.001$), followed by ADHD ($X^2 = 14.785$; $p < 0.001$), anxiety ($X^2 = 12.088$; $p < 0.005$), and OCD ($X^2 = 7.214$; $p < 0.001$). Its associations with tic severity ($X^2 = 5.097$; $p < 0.05$) and OCB were very weak ($X^2 = 4.879$; $p < 0.05$). Accordingly, sleeping problems were significantly more common in the "TS + comorbidity" group [$n = 256$ (28.7%)] compared to patients with TS only [$n = 4$ (5.3%); $Z = 4.3905$; $p < 0.01$]. There was also a significant positive association between the presence of sleeping problems and the comorbidity score ($X^2 = 53.569$; $p < 0.001$).

Anxiety

The clinical lifetime diagnosis of any kind of an anxiety disorder was made in 323 patients (31.4%). Prevalence rates for anxiety were significantly higher amongst those with TS+OCD (55.6%, $n = 45$) compared to those with TS+ADHD (16.8%, $n = 102$; $Z = 4.794$; $p < 0.01$; refer to **Table 4**).

Depression

The lifetime diagnosis of depression was made in 236 patients (22.9%). However, depression was found in 55.6% of the patients with TS+OCD, but only in 9.9% of the TS+ADHD group, resulting in a significant difference ($Z = 5.988$; $p < 0.01$; refer to **Table 4**). A significant positive association was also found between the prevalence of depression and anxiety ($X^2 = 69.083$; $p < 0.001$).

SIB

Lifetime prevalence of SIB was reported by 405 (39.4%) patients. SIB was found to have a significant positive associations with the presence of complex motor tics ($X^2 = 57.551$; $p < 0.01$), OCB ($X^2 = 32.026$; $p < 0.01$), and anxiety ($X^2 = 31.634$; $p < 0.01$), in a descending order of the strength of the association (refer to **Table 4**). For association with coprophenomena, see above.

Sub-Classification of TS

For the purpose of further exploring Robertson and Cavanna's (2007) sub-classification of TS and common comorbidities, the three clusters provided by them were examined for expected differences in certain variables: (i) cluster 1, which consists of only tics ("TS only"; $n = 75$), (ii) cluster 2, which consists of TS and comorbid ADHD and rage attacks ($n = 56$), and (iii) cluster 3, which consists of TS and comorbid OCD, OCB, anxiety, depression, and SIB ($n = 209$). Since we included only those patients in clusters 2 and 3 who had all of the aforementioned comorbidities (e.g., cluster 2 consisted of TS patients with both comorbid ADHD and rage attacks, and not just one of the two), only one-third of the total number of patients could be classified using Robertson and Cavanna's (2007) sub-classification criteria. The rate of copropraxia in cluster 2 was 16.1 vs. 4.8% in cluster 3 and 2.7% in cluster 1, the difference between cluster 2 and cluster 3 being significant in the direction of the hypothesis ($X^2 = 25.887$; $p < 0.001$). The ability for tic suppression in the "TS only" group was 91.7%, and was significantly higher than that in cluster 2 (78.2%) ($Z = 2.1983$; $p < 0.05$), but not significantly different from that in cluster 3 (89.7%) ($Z = 0.5004$; n.s.). The percentage of patients reporting PU based on the different clusters was the following: cluster 1 ("TS only"): 57.7%, cluster 2: 62.3%, and cluster 3: 75.1%. PU reported in cluster 3 was significantly higher than that reported in cluster 1 ($Z = 2.835$; $p < 0.001$) but was not significantly higher than PU reported in cluster 2 ($Z = 1.9029$; n.s.).

Gender Differences

The male-to-female ratio in the current TS group is 3.4:1. **Table 9** shows results of gender-based comparisons of TS-related phenomena from data obtained from all patients in the sample demonstrating that males suffer more often from coprolalia, copropraxia, palilalia, OCB, hyperactivity, and inattention. They are also more likely to experience PU. Females, on the other hand, are more likely than males to suffer from sleeping problems. No significant gender differences were found with regards to age at onset of both tic types in general, [$t_{(963)} = 0.022$; n.s.], MT [$t_{(542)} = -1.216$; n.s.], and PT [$t_{(951)} = 1.489$; n.s.]. No significant relationship was found between comorbidity score and gender [$t_{(1025)} = 1.398$; n.s.].

DISCUSSION

In this study, we report clinical information collected from a large sample of 1032 patients with different forms of primary tic disorder. As two of the primary objectives of this study were the exploration of the TS spectrum and the verification of the accuracy of the sub-group classifications propounded by other researchers, we concentrated on TS in association to its various comorbidities.

Age at Onset and Tic Severity

The mean age at onset of tics in general, and MT and PT in particular, were all found to be consistent with findings of other studies (Freeman et al., 2000), but somewhat above the data in TS literature. This could be because there was a high number of adult participants in our study and since such data are always

TABLE 9 | Gender differences in various TS-related aspects.

	Males *n* (%)	Mean STSS-GSR	Females *n* (%)	Mean STSS-GSR	*Z*-Score	*p*-value	Significance	Dominant Gender
Coprophenomena	242 (30.4)	3.4	48 (20.3)	3.35	3.0205	0.002	$p < 0.01$	m > f
-Coprolalia	205 (25.9)	3.48	42 (17.8)	3.36	2.5161	0.01	$p < 0.05$	m > f
-Copropraxia	141 (17.8)	3.45	19 (8.1)	3.83	3.6019	0.0003	$p < 0.01$	m > f
Palilalia	278 (35.1)	3.19	68 (25.8)	3.35	1.7465	0.08	$p < 0.01$	m > f
Echolalia	231 (29.1)	3.22	59 (25)	3.18	1.2067	0.22	n.s.	–
Echopraxia	183 (23.1)	3.19	55 (23.3)	3.22	−0.1009	0.92	n.s.	–
OCD	72 (9.1)	3.04	31 (13.1)	3.41	−1.8411	0.06	n.s.	–
OCB	509 (64)	2.91	128 (54.2)	2.94	2.6946	0.007	$p < 0.01$	m > f
Compulsions	552 (69.7)	2.83	153 (65.1)	2.97	1.3097	0.19	n.s.	–
-Non Just Right Experiences	446 (56.2)	2.94	129 (54.7)	2.06	0.3719	0.71	n.s.	–
-Ordering	185 (23.3)	3.18	53 (22.5)	3.17	0.251	0.80	n.s.	–
-Checking	281 (35.5)	3.08	81 (34.3)	3.14	0.2769	0.77	n.s.	–
-Counting	97 (12.3)	3.03	28 (11.9)	3.41	0.133	0.89	n.s.	–
-Washing	66 (8.3)	2.72	20 (8.5)	3.05	−0.0894	0.92	n.s.	–
Obsessions	273 (34.6)	3.02	84 (35.7)	2.93	−0.3115	0.75	n.s.	–
Hyperactivity	294 (31.4)	2.97	42 (17.9)	2.97	5.51	<0.0000	$p < 0.01$	m > f
Inattention	337 (42.4)	2.9	68 (29.1)	3.03	3.5378	0.0004	$p < 0.01$	m > f
ADHD	387 (48.6)	2.92	76 (32.2)	2.92	4.4449	<0.0000	$p < 0.01$	m > f
Sleeping problems	198 (25.1)	2.97	75 (32.1)	3.11	−2.1122	0.03	$p < 0.05$	f > m
Anxiety	246 (31)	3.06	7 (32.8)	3.03	−0.5012	0.60	n.s.	–
Depression	177 (22.3)	3.1	59 (25)	3.32	−0.8879	0.37	n.s.	–
Rage attacks	460 (58)	2.93	134 (57)	3.07	0.2755	0.77	n.s.	–
SIB	310 (38.9)	3.2	97 (41.1)	3.15	−0.5955	0.54	n.s.	–
Severity of Tics* (severe)	67 (8.7)	–	28 (12.2)	–	−1.6104	0.10	n.s.	–
Premonitory urges	551 (72.3)	2.85	149 (65.1)	2.99	2.133	0.03	$p < 0.05$	m > f
Tic suppression	655 (85.2)	2.82	198 (86.1)	2.85	−0.3439	0.72	n.s.	–

**according to STSS-GSR, severe, 5; very severe, 6 (range, 0–6).*
OCD, obsessive-compulsive disorder; OCB, obsessive-compulsive behavior; ADHD, attention-deficit hyperactivity disorder; SIB, self-injurious behavior; TS, Tourette Syndrome; STSS-GSR, Global Severity Rating of the Shapiro Tourette-Syndrome Severity Scale; n.s., not significant.

based on patient reports and memory, it is possible that there was a recollection bias toward slightly greater age at onset, because tic onset goes back many years, and thus could not be dated precisely.

We found no association between tic severity and age at onset, which is contradictory to previous research (Khalifa and von Knorring, 2005). To the best of our knowledge, there is almost no data available investigating specifically the association between age at tic onset and tic severity, although this is an important clinical question that is often asked by parents of affected children. From our results, it is strongly—and for the first time—suggested that there is no such association, although one could easily expect this. This finding is in line with results of the large clinical database by Freeman et al. (2000): although not specifically mentioned, it can be concluded that in this dataset there was also no association between age at tic onset and tic severity, since they found no difference with respect to age at tic onset depending on the presence of comorbidities (but more severe tics in patients with comorbidities compared to those without). When looking at the data by Khalifa and von Knorring (2005) in more detail, the suggested correlation between tic severity and age at onset appears questionable, since this was found only in the very small group of patients with TS ($n = 25$), but not in the larger group diagnosed with chronic motor/vocal tics ($n = 58$).

We also found that for a majority of the participants in our study, tic onset was much earlier than the age of 18 (refer to **Table 2**). This is consistent with Freeman et al.'s (2000) finding, where in 92.7% of the sample, tic onset was before 10 years of age, and in 99% of the sample, it was before 16 years. Thus, data from these two large datasets clearly confirm that the "18-year maximum age of onset criterion" used in DSM-5 is well-founded and should be maintained. In our sample, the median for tic severity was found to be mild to moderate (median STSS-GSR = 3; 25th percentile = 2, 75th percentile = 4), which demonstrates how rare extremely severe TS is, since even in a highly specialized outpatient unit such as our center, 904 (87.6%) patients had mild to moderate tics and only 95 (9.2%) had severe or very severe tics. Our data do not demonstrate the well-known age dependency of tic severity, but a continuous tic increase with increasing age. However, this should be interpreted as a bias stemming from the fact that this was a cross-sectional and not a longitudinal study, tic assessment was done only once during the first medical examination, and patients tend to come to the clinic when their

tics get worse and usually not when their tics reduce. At our center several patients present for the first only at adult age (with or without having received the correct diagnosis of TS before). It can be assumed that the majority of these patients belong to the small group of those patients who suffer not only from persistent tics, but also from more severe tics. This selection bias may be the reason why we failed to demonstrate the well-known age dependency of tics.

Completely in line with available data (Freeman et al., 2000), tic severity was positively related to the comorbidity score in that greater the number of comorbidities, higher the tic severity, and also that those with one or more comorbidities had significantly more severe tics than those without comorbid disorders. Comorbid SIB was found to have the highest impact on tic severity, followed by rage attacks, depression, anxiety, OCD, and lastly, ADHD. In line with our hypothesis and TS literature (Lebowitz et al., 2012), we also found that tic severity was significantly greater in those with comorbid OCD but no ADHD than in those with comorbid ADHD but no OCB/OCD. These findings further corroborate the hypothesis that TS plus OCD is a more severe form of TS, that OCB/OCD is part of the TS spectrum, and that ADHD should be better conceptualized as a separate problem (Trillini and Müller-Vahl, 2015).

Coprophenomena, Echophenomena, and Paliphenomena

The prevalence rates of both coprolalia and copropraxia were higher as compared to the percentages reported in Freeman et al.'s study on coprophenomena, where the rate of coprolalia ever was 18.5% and that of copropraxia was 5.7% (Freeman et al., 2009), but were lower than those reported by Cavanna et al. (2011), in which coprolalia and copropraxia were reported by 30.4 and 21.1% of the sample, respectively. The relatively high prevalence rates reported in our study are possible due to the fact that ours is a highly specialized clinic, where mostly patients with several complex tics are referred. In addition, almost half of our sample comprised adult patients. This might be another reason for the higher prevalence rates of coprophenomena in our sample, since the prevalence of coprophenomena increases with age. Finally, all patients were specifically asked for the presence of copro-, echo-, and paliphenomena.

The presence of all, coprophenomena, echophenomena, and paliphenomena were associated with both tic severity and the number of comorbidities, corroborating the hypothesis that "full blown TS" is a more severe form of TS as suggested by Robertson and Baron-Cohen (1998).

We also found that for both copro- and echophenomena, the prevalence of the respective PT form (i.e. copro- and echolalia) was higher than their corresponding MT form (copro- and echopraxia). This is well-known (Freeman et al., 2000, 2009) and suggests that copro- and echophenomena are, in a way, different from simple tics, in which the frequency is vice-versa (MT > PT). Assuming that tics present "fragments of innate behavioral routines" as suggested recently (Leckman et al., 2013), it can be speculated that copro-/echolalia are more common than copro-/echopraxia, because swearing and repeating noises (such as coughing) is nearer to "normal behavior" than performing obscene gestures and the repetition of movements. However, and in contrast, excessive brief movements such as eye blinking and grimacing are "nearer to normal" than excessive fast and meaningless noises.

In agreement with results from Freeman et al.'s database (2009), we found that both coprolalia and copropraxia were most highly associated with SIB, while associations with other comorbidities were much weaker. The strong association of coprophenomena with SIB, along with the equally high association between SIB and complex motor tics suggests that SIB in TS belongs to the coprophenomena spectrum and is a complex tic rather than an OC symptom, as has previously been suggested (Mathews et al., 2004). However, Mathews et al. (2004) found a correlation between OC symptoms and "mild SIB," while "severe SIB" correlated with tic severity and, therefore, they suggested the presence of two distinct forms of SIB. In this study, no distinction was made with respect to severity in a mild form (including hand banging, compulsive skin picking, self-hitting, and lip biting) and a severe form with serious injuries. In line with Mathews et al.'s results (2004), we found no strong association between SIB and rage attacks. Our finding that SIB had a higher impact on tic severity than all other comorbidities (rage attacks, depression, anxiety, OCD, and ADHD), in addition with the well-known correlation between coprophenomena and tic severity (Freeman et al., 2009), further suggests that SIB is a specific form of a complex tic rather than an OC symptom. Our findings may have important implications for the clinical management of SIB and suggest the use of both behavioral therapy and antipsychotics instead of serotonin reuptake inhibitors.

Prevalence rates of echolalia and echopraxia were found to be lower than the rates reported by Eapen et al. in their 2004 paper (echolalia: 37.4%, $n = 34$; echopraxia: 29.9%, $n = 27$) as well as those reported by Cavanna et al. (2011) [echolalia: 40.3%; echopraxia: 36.9%; (numbers not provided)], but higher compared to other samples: echolalia: 16% ($n = 32$; Cardoso et al., 1996) and echolalia: 21% and echopraxia: 18% ($n = 71$; Neal and Cavanna, 2013). In our sample, the prevalence rate for palilalia was found to be similar to that in Eapen et al.'s (2004) sample (29.7%) as well as to Cavanna et al.'s (2011) sample (31.7%). These differences may be due to the fact that the prevalence rate for echophenomena and palilalia in any study depends on whether the examiner asks the patient for relevant symptoms, or only records what (s)he observes in the patient. Furthermore, the way in which these questions are framed by the examiner may also affect the prevalence rate. In the case of echophenomena, the examiner in the present study specifically asked all patients "when you see movements and hear noises from other people—independent of whether these are tics or normal movements, or noises like coughing, or even noises from animals or machines—have you ever felt that you had to imitate this?" Only when the patient responded in the affirmative and said that (s)he had acted accordingly was (s)he recorded as positive for the echophenomenon. In any case, our data confirm the assumption that echophenomena are a core symptom of TS (Ganos et al., 2012).

Premonitory Urges and Tic Suppression

Banaschewski et al. (2003) conducted a study to determine if there is any age dependency of the presence of PU as well as of tic suppression (refer to **Table 7**). In our study, we aimed to replicate these findings. Our results were mixed in terms of support for Banaschewski et al.'s findings. In general, PU and tic suppression showed a clear age dependency. Secondly, although PU and the ability to suppress tics were strongly associated, in each age group, the percentage of patients who were capable of tic suppression was greater than the percentage of those who experienced a PU, indicating that tic suppression is possible without necessarily being aware of a PU. However, there were also some relevant differences between our results and those of Banaschewski et al. Firstly, our results did not show "jumps" for the development of either PUs or the ability to suppress tics, unlike Banaschewski et al.'s findings, which suggested that children develop the ability for tic suppression at around the age of 10 years, and for PU around age 14. Secondly and most important, the percentages of those patients who experience a PU as well as those who could suppress their tics were much higher at each age group compared to Banaschewski et al.'s study. We found that both PU and tic suppressing ability is already present in a high number of TS patients before the age of 8 (refer to **Table 7**)—much earlier than previously thought. This indicates that PU and suppression exist right from the beginning, and do not develop during the course of TS. From our data, therefore, it is suggested that the only reason that PU and tic suppression cannot be seen in (very) young children is because they are unable to either introspect or express them well-enough. This has important implications with respect to the treatment with habit reversal training and is in line with efficacy of this treatment even in younger children as was demonstrated by Piacentini et al. (2010).

On considering the age dependency based on the natural history as described by Bloch et al. (2006), we found high percentages of those who experienced PU and had the ability to suppress tics at every age category. Here too, we observed a clear age dependency, with a consistent increase of around 12–20% at every successive age category.

Finally, our results also show that the higher the tic severity, the more likely it is that the patient experiences a PU. However, no such association was observed between tic severity and tic suppression ability. With respect to Robertson and Cavanna's (2007) classification, we found that patients in cluster 3 (comorbid OCD/anxiety/depression/SIB) were almost equally able to suppress their tics compared to those in cluster 1 ("TS only"). However, a significant difference exists in tic suppression between patients with TS only and patients in cluster 2 (comorbid ADHD/rage attacks). This suggests that tic suppression is independent from comorbid OCD/anxiety/depression/SIB, but patients who suffer from ADHD are less able to suppress their tics. Results also showed that PU was higher in the "TS + comorbidity" group than in the "TS only" group, which is supported by past findings (Eddy and Cavanna, 2013). Particularly, it was highest in cluster 3 (including patients with OCD/OCB/SIB/anxiety/depression), followed by cluster 2 (including patients with ADHD/rage attacks) and finally by the group of patients having no comorbidities (cluster 1, "TS only"). This too is in line with Eddy and Cavanna's (2013) finding that comorbidities such as anxiety, OCD, and ADHD are strongly correlated with PU. Finally, our results also showed a strong positive association between PU and "not just right experiences" (and to a relatively lesser degree with OCB, but not OCD), which indicates that PU may be a form of this specific and very common form of OCB in TS. This further corroborates the hypothesis that "not just right experiences" are intrinsic to the phenomenology of TS (Neal and Cavanna, 2013).

Influence of Comorbidities

The fact that only a small percentage of the TS sub-group had not reported or were diagnosed with any comorbidity is consistent with relevant literature (Freeman et al., 2000; Khalifa and von Knorring, 2006; Cavanna et al., 2011; Robertson et al., 2015). However, relative to the total TS population, this could be a bias arising out of the method of data collection; the sample is a clinical one and mostly, only patients with more severe tics and more comorbidities come for a referral. This could be because the quality of one's life is impaired by common comorbidities of TS far more than the tics themselves, as has been consistently demonstrated in past literature (Pringsheim et al., 2009; Müller-Vahl et al., 2010; Jalenques et al., 2012). Research has shown that quality of life (Eddy et al., 2012) and psychosocial health (Pringsheim et al., 2009) was most adversely affected on all domains in those TS patients suffering from both comorbid OCD and comorbid ADHD, whereas having only one of the two comorbidities causes domain-specific impairment. Rizzo et al. (2014) found a significantly lower quality of life among those patients having TS+OCD/TS+ADHD/TS+OCD+ADHD as compared to patients with pure TS. Studies have also found comorbid depression to be one of the most significant independent factors impairing the quality of life of TS patients (Müller-Vahl et al., 2010; Jalenques et al., 2012).

In our sample, rage attacks were found to be the most common comorbidity followed by ADHD. In contrast to this, Freeman et al. (2000) found ADHD (60%) to be the most highly occurring comorbidity. With respect to anxiety, depression, and SIB, the sample in the current study showed much higher prevalence rates compared to Freeman et al.'s sample, where 18, 12.1, and 14% had anxiety, depression, and SIB respectively. All these differences in prevalence rates of different comorbidities can probably be best explained by age differences between our sample and Freeman et al.'s sample (mean age = 18.9 vs. 13.2 years), as the older the patients, the more likely it is that they have comorbidities such as depression and sleeping problems and the younger they are, the more likely it is that they have ADHD. The prevalence rate of depression was also much higher than the 6.0% found by Khalifa and von Knorring (2006), but this could be because their sample consisted only of school children in the 7–15 years age range. Our results, however, were very similar to the 27.8% reported by Robertson et al. (2006). Cavanna et al. (2009), in their review of the literature on the phenotype of TS stated that among the 5295 patients with TS who presented at specialist clinics, the prevalence rate of depressive symptomatology was found to range from 13 to 76%.

With respect to OC symptoms, we found a lower prevalence rate for OCD compared to the Freeman et al.'s (2000) sample in their 2000 paper (27%). This might be explained by the fact that the evaluator (KMV) was extremely particular whilst diagnosing OCD and often made the diagnosis of OCB, unless all symptoms matched the prerequisites. However, the by far most often reported OC symptom in both groups OCD and OCB was a "non-just right experience" (refer to **Table 8**), which corroborates recent findings suggesting that "not just right experiences" are intrinsic to the clinical phenomenology of TS (Neal and Cavanna, 2013). As described above, in this context again it is worth mentioning that from our findings it is suggested that premonitory urges (PU) prior to the occurrence of tics may also be a form of a "non-just right experience." In addition, we found that among those who had either OCD or OCB in our sample, compulsions such as checking and ordering were much higher, whereas those of counting and washing tended to be lower. This is in line with past research in TS related OCD/OCB (Worbe et al., 2010). According to Stewart et al. (2008), there are four factors for OCD: factor 1 (aggressive/sexual/religious/somatic/checking); factor 2 (symmetry/ordering/counting/repeating); factor 3 (contamination/cleaning), and factor 4 (hoarding). Of these, only factor 2 was found to be common in TS, which further supports the hypothesis that OCD/OCB in TS is different form pure OCD.

We found that almost half the total number of patients diagnosed with rage attacks did not have either hyperactivity or inattention, which is consistent with the finding that rage attacks, as compared to hyperactivity and inattention, is highly common and represents one symptom of the typical spectrum of disinhibited behaviors in TS that occurs in a substantial number of patients independently from the presence of ADHD (Frank et al., 2011). The assumption that rage attacks in TS are slightly separate from and independent of the ADHD spectrum is further supported by our finding that in our sample both inattention and hyperactivity, but not rage attacks were more common in males than in females.

Sleeping problems among the patients in our sample were very common, and were found to be about five times as common in the "TS + comorbidity" group as compared to the "TS only" group. Such problems were most highly associated with depression, indicating the well-stated finding that in TS depression has a strong adverse effect on the patients' quality of life (Müller-Vahl et al., 2010; Eddy et al., 2011; Jalenques et al., 2012). Other comorbidities (such as ADHD, anxiety, and OCD, in a descending order of the strength of the association) as well as tics (with lesser strength of association) may also have an impact, but these associations were found to be much weaker in comparison to depression. The strong association between sleeping problems and depression has important clinical implication: TS patients suffering from sleeping problems should be screened for depression and, if necessary, treated for this disorder. We found sleeping problems in the comorbid group as twice as common in the comorbid group studied by Freeman et al. (2000). This can be explained by the age difference between both samples with more children in the Freeman et al.'s group and the strong association with depression. Significantly higher prevalence rates of both anxiety and depression in the TS+OCD group as compared to the TS+ADHD group are in line with previous research findings (Lebowitz et al., 2012; Trillini and Müller-Vahl, 2015) and also provide support to our hypothesis that TS is more closely related with OCD, anxiety, and depression as compared to ADHD.

Influence of Gender

The male-to-female ratio in our sample was 3.4:1 and therefore comparable to several other studies (Erenberg et al., 1986; Freeman et al., 2000). In addition, results showed significant predominance of males over females with respect to coprophenomena (coprolalia, copropraxia, and overall), OCB, hyperactivity, inattention, ADHD, and PU. A significantly greater percentage of females in the sample had sleeping problems. In contrast to our findings, past research in non-TS samples has consistently shown twofold higher prevalence of anxiety and depression in females over males (Bijl et al., 2002). In agreement with recent studies in larger samples in both children (Robertson et al., 2006) and mixed groups (Freeman et al., 2000), we did not find gender differences with respect to anxiety and depression. In contrast, in a recent internet-based survey ($n = 460$ adults) women reported more often about both depressive symptoms and non-OCD anxiety disorders (35.6 and 33.7%, respectively) compared to men (23.2 and 15.5%, respectively; Lewin et al., 2012). However, these results should be interpreted with caution not only due to the well-known limitations related to such a study design, but also other findings (e.g., insignificantly higher prevalence rate for self-reported ADHD in female compared to male). Thus, our results as well as past research (Freeman et al., 2000; Robertson et al., 2006) suggest that the nature of both depression and anxiety disorders in patients with TS differs from that found in the general population. These disorders are a part of the TS spectrum and are not only secondary due to tics and an impaired quality of life. This, in addition, also supports the assumption that the TS subgroup with comorbid OCD, anxiety, and depression represents a more severe form of the disease.

We found no gender differences in the prevalence of rage attacks. Since there is a general predominance of males over females with respect to the prevalence of ADHD in ADHD-only samples (Kessler et al., 2006), TS+ADHD samples (Freeman et al., 2000), as well as in the current study sample, our finding of similar prevalence rates of rage attacks for both genders is in line with relevant literature (Frank et al., 2011) which shows that in TS, rage attacks are independent and separate from ADHD.

Limitations

Our study has some noticeable limitations. Primarily, there was an absence of a standardized assessment procedure and diagnoses were made based on judgments of a single expert clinician, thereby increasing the likelihood of errors caused by various biases in evaluation. However, KMV is a highly trained expert in both neurology and psychiatry and thus had a rich experience in diagnosing movement disorders from the very start of her career. Since most studies are carried out by clinicians with less expertise in diagnosing TS, we do

not see this is being any major disadvantage of our study. Another limitation is a lack of temporal stability, since data was collected over almost two decades and the diagnoses were made based on the version of the DSM valid at that time. It should be noted though, that the core criteria of tic disorders did not change over time, therefore this should not be too much of a concern. The cross-sectional nature of our data also makes it difficult to learn about the temporal progression of tic severity. Lastly, the possible effects of various medications on tic suppression were not controlled for. It can be assumed that using medication would improve one's ability to suppress tics, and therefore we cannot dismiss the idea that its use by some of the patients influenced our results. However, to the best of our knowledge, there is no study that looks particularly into such an influence.

CONCLUSION

We believe that this study is the first single-site study on TS and TS-related phenomena involving such a large sample. For the first time, our results suggest that an early age of tic onset is not necessarily associated with higher tic severity at subsequent ages.

Secondly, it has revealed that PU and tic suppression are factors that are present from earlier than previously assumed in past literature (Banaschewski et al., 2003). Both phenomena seem to emerge in parallel to the tics rather than later in the course of the disorder. Therefore, our data corroborate more recent findings suggesting that PU is not a prerequisite for tic suppression.

Thirdly, our data suggests that PU could represent of specific type of OCB and in particular a "not just right experience."

Fourthly, our data suggest that SIB belongs to the coprophenomena spectrum and hence should be conceptualized as a complex tic rather than a compulsion. This has important implications for the treatment of severe SIB.

Fifthly, our results suggest that both depression and anxiety disorders in patients with TS differ from their presentation in non-TS samples. Sleeping problems are common in (adult) patients with TS and are most often caused by comorbid depression. Therefore, patients with TS reporting about sleeping problems should be screened for comorbid depression.

Sixthly, rage attacks represent a typical symptom of disinhibited behaviors in patients with TS. For clinicians it is important to classify rage attacks as a manifestation of the disease that often occur even in the absence of comorbid ADHD.

Seventhly, patients with TS and comorbid ADHD are less able to suppress their tics when compared with those without attention deficits.

Finally, our data demonstrate that TS+OCD is a more severe form of the disease. Furthermore, our clinical data adds to other clinical observations that subjects with ADHD may not be able to suppress tics as well as those without ADHD. Synthesizing all our data, we find support for the hypothesis that comorbid OCD/OCB, depression, and anxiety belong to the TS spectrum, while ADHD needs to be considered an independent diagnosis.

AUTHOR CONTRIBUTIONS

TS engaged mainly data analysis, data interpretation, writing of the manuscript. EJ provided advice on statistical analysis, and participated in manuscript preparation. KM managed data collection, formulated the study design, supervised data analysis and manuscript preparation.

ACKNOWLEDGMENTS

We wish to thank Lars Makowski, Sarah Bottor, and Anna-Sophie Ellinghausen for helping us build up the extensive database used in our study. Special thanks also to all the participants for their effort.

REFERENCES

American Psychiatric Association (2013). *Diagnostic and statistical manual of mental disorders, 5th Edn*. Washington, DC.

Banaschewski, T., Woerner, W., and Rothenberger, A. (2003). Premonitory sensory phenomena and suppressibility of tics in Tourette syndrome: developmental aspects in children and adolescents. *Dev. Med. Child Neurol.* 45, 700–703. doi: 10.1017/s0012162203001294

Bijl, R. V., De Graaf, R., Ravelli, A., Smit, F., and Vollebergh, W. A. (2002). Gender and age-specific first incidence of DSM-III-R psychiatric disorders in the general population. *Soc. psychiatry psychiatr. Epidemiol.* 37, 372–379. doi: 10.1007/s00127-002-0566-3

Bloch, M. H., Peterson, B. S., Scahill, L., Otka, J., Katsovich, L., Zhang, H., et al. (2006). Adulthood outcome of tic and obsessive-compulsive symptom severity in children with Tourette syndrome. *Arch.Pediatr. Adolesc. Med.* 160, 65–69. doi: 10.1001/archpedi.160.1.65

Cardoso, F., Veado, C. C., and de Oliveira, J. T. (1996). A Brazilian cohort of patients with Tourette's syndrome. *J. Neurol. Neurosurg. Psychiatry* 60, 209–212. doi: 10.1136/jnnp.60.2.209

Cavanna, A. E., Critchley, H. D., Orth, M., Stern, J. S., Young, M. B., and Robertson, M. M. (2011). Dissecting the Gilles de la Tourette spectrum: a factor analytic study on 639 patients. *J. Neurol. Neurosurg. Psychiatry* 82, 1320–1323. doi: 10.1136/jnnp.2010.225029

Cavanna, A. E., Servo, S., Monaco, F., and Robertson, M. M. (2009). The behavioral spectrum of Gilles de la Tourette syndrome. *J. Neuropsychiatry Clin. Neurosci.* 21, 13–23. doi: 10.1176/jnp.2009.21.1.13

Cubo, E., Gabriely Galán, J. M., Villaverde, V. A., Velasco, S. S., Benito, V. D., Macarrón, J. V., et al. (2011). Prevalence of tics in schoolchildren in central Spain: a population-based study. *Pediatr. Neurol.* 45, 100–108. doi: 10.1016/j.pediatrneurol.2011.03.003

Eapen, V., Fox-Hiley, P., Banerjee, S., and Robertson, M. (2004). Clinical features and associated psychopathology in a Tourette syndrome cohort. *Acta Neurol. Scand.* 109, 255–260. doi: 10.1046/j.1600-0404.2003.00228.x

Eddy, C. M., and Cavanna, A. E. (2013). Premonitory urges in adults with complicated and uncomplicated Tourette syndrome. *Behav. Modif.* 8, 264–275. doi: 10.1177/0145445513504432

Eddy, C. M., Cavanna, A. E., Gulisano, M., Agodi, A., Barchitta, M., Calì, P., et al. (2011). Clinical correlates of quality of life in Tourette syndrome. *Mov. Disord.* 26, 735–738. doi: 10.1002/mds.23434

Eddy, C. M., Cavanna, A. E., Gulisano, M., Calì, P., Robertson, M. M., and Rizzo, R. (2012). The effects of comorbid obsessive-compulsive disorder and attention-deficit hyperactivity disorder on quality of life in tourette syndrome. *J. Neuropsychiatry Clin. Neurosci.* 24, 458–462. doi: 10.1176/appi.neuropsych.11080181

Erenberg, G., Cruse, R. P., and Rothner, A. D. (1986). Tourette syndrome: an analysis of 200 pediatric and adolescent cases. *Cleve Clin. Q.* 53, 127–131. doi: 10.3949/ccjm.53.2.127

Frank, M. C., Piedad, J., Rickards, H., and Cavanna, A. E. (2011). The role of impulse control disorders in Tourette syndrome: an exploratory study. *J. Neurol. Sci.* 310, 276–278. doi: 10.1016/j.jns.2011.06.032

Freeman, R. D., Fast, D. K., Burd, L., Kerbeshian, J., Robertson, M. M., and Sandor, P. (2000). An international perspective on Tourette syndrome: selected findings from 3500 individuals in 22 countries. *Develop. Med. Child Neurol.* 42, 436–447. doi: 10.1017/S0012162200000839

Freeman, R. D., Zinner, S. H., Müller-Vahl, K. R., Fast, D. K., Burd, L. J., Kano, Y., et al. (2009). Coprophenomena in Tourette syndrome. *Develop. Med. Child Neurol.* 51, 218–227. doi: 10.1111/j.1469-8749.2008.03135.x

Ganos, C., Ogrzal, T., Schnitzler, A., and Münchau, A. (2012). The pathophysiology of echopraxia/echolalia: relevance to Gilles de la Tourette syndrome. *Mov. Disord.* 27, 1222–1229. doi: 10.1002/mds.25103

Hirschtritt, M. E., Lee, P. C., Pauls, D. L., Dion, Y., Grados, M. A., Illmann, C., et al. (2015). Lifetime prevalence, age of risk, and genetic relationships of comorbid psychiatric disorders in Tourette syndrome. *JAMA Psychiatry* 72, 325–333. doi: 10.1001/jamapsychiatry.2014.2650

Jalenques, I., Galland, F., Malet, L., Morand, D., Legrand, G., Auclair, C., et al. (2012). Quality of life in adults with Gilles de la Tourette Syndrome. *BMC psychiatry* 12:109. doi: 10.1186/1471-244X-12-109

Kessler, R. C., Adler, L., Barkley, R., Biederman, J., Conners, C. K., Demler, O., et al. (2006). The prevalence and correlates of adult ADHD in the United States: results from the National Comorbidity Survey Replication. *Am. J. Psychiatry* 163, 716–723. doi: 10.1176/ajp.2006.163.4.716

Khalifa, N., and von Knorring, A. L. (2005). Tourette syndrome and other tic disorders in a total population of children: clinical assessment and background. *Acta Paediatr.* 94, 1608–1614. doi: 10.1111/j.1651-2227.2005.tb01837.x

Khalifa, N., and von Knorring, A. L. (2006). Psychopathology in a Swedish population of school children with tic disorders. *J. Am. Acad. Child Adolesc. Psychiatry* 45, 1346–1353. doi: 10.1097/01.chi.0000251210.98749.83

Lebowitz, E. R., Motlagh, M. G., Katsovich, L., King, R. A., Lombroso, P. J., Grantz, H., et al. (2012). Tourette syndrome in youth with and without obsessive compulsive disorder and attention deficit hyperactivity disorder. *Eur. Child Adolesc. Psychiatry* 21, 451–457. doi: 10.1007/s00787-012-0278-5

Leckman, J. F., Bloch, M. H., Sukhodolsky, D. G., Scahill, L., and King, R. A. (2013). "Phenomenology of tics and sensory urges: the self under siege," in *Tourette Syndrome*, ed D. Martino (New York, NY: Oxford University Press), 3–25.

Leckman, J. F., Walker, D. E., and Cohen, D. J. (1993). Premonitory urges in Tourette's syndrome. *Am. J. Psychiatry* 150, 98–102. doi: 10.1176/ajp.150.1.98

Leckman, J. F., Zhang, H., Vitale, A., Lahnin, F., and Lynch, K., Bondi, C., et al. (1998). Course of tic severity in Tourette syndrome: the first two decades. *Pediatrics* 102, 14–19. doi: 10.1542/peds.102.1.14

Lewin, A. B., Murphy, T. K., Storch, E. A., Conelea, C. A., Woods, D. W., Scahill, L. D., et al. (2012). A phenomenological investigation of women with Tourette or other chronic tic disorders. *Compr. Psychiatry* 53, 525–534. doi: 10.1016/j.comppsych.2011.07.004

Mathews, C. A., Waller, J., Glidden, D., Lowe, T. L., Herrera, L., Budman, C., et al. (2004). Self injurious behaviour in Tourette syndrome: correlates with impulsivity and impulse control. *J. Neurol. Neurosurg. Psychiatry* 75, 1149–1155. doi: 10.1136/jnnp.2003.020693

Müller-Vahl, K., Dodel, I., Müller, N., Münchau, A., Reese, J. P., Balzer-Geldsetzer, M., et al. (2010). Health-related quality of life in patients with Gilles de la Tourette's syndrome. *Mov. Disord.* 25, 309–314. doi: 10.1002/mds.22900

Neal, M., and Cavanna, A. E. (2013). "Not just right experiences" in patients with Tourette syndrome: complex motor tics or compulsions?. *Psychiatry Res.* 210, 559–563. doi: 10.1016/j.psychres.2013.06.033

Packer, L. E. (1997). Social and educational resources for patients with Tourette syndrome. *Neurol. Clin.* 15, 457–473. doi: 10.1016/S0733-8619(05)70326-1

Piacentini, J., Woods, D. W., Scahill, L., Wilhelm, S., Peterson, A. L., Chang, S., et al. (2010). Behavior therapy for children with Tourette disorder: a randomized controlled trial. *JAMA* 303, 1929–1937. doi: 10.1001/jama.2010.607

Pringsheim, T., Lang, A., Kurlan, R., Pearce, M., and Sandor, P. (2009). Understanding disability in Tourette syndrome. *Develop. Med. Child Neurol. 51,* 468–472. doi: 10.1111/j.1469-8749.2008.03168.x

Rizzo, R., Gulisano, M., Pellico, A., Calì, P. V., and Curatolo, P. (2014). Tourette syndrome and comorbid conditions a spectrum of different severities and complexities. *J. Child Neurol.* 29, 1383–1389. doi: 10.1177/0883073814534317

Robertson, M. M. (1994). Annotation: Gilles De La Tourette Syndrome—an update. *J. Child Psychol. Psychiatry* 35, 597–611. doi: 10.1111/j.1469-7610.1994.tb01209.x

Robertson, M. M. (2000). Tourette syndrome, associated conditions and the complexities of treatment. *Brain* 123, 425–462. doi: 10.1093/brain/123.3.425

Robertson, M. M., and Baron-Cohen, V. (1998). *Tourette Syndrome: The Facts, 1st Edn.* London: Oxford Press.

Robertson, M. M., and Cavanna, A. E. (2007). The Gilles de la Tourette syndrome: a principal component factor analytic study of a large pedigree.*Psychiatr. Genet.* 17, 143–152. doi: 10.1097/YPG.0b013e328015b937

Robertson, M. M., Cavanna, A. E., and Eapen, V. (2015). Gilles de la Tourette syndrome and disruptive behavior disorders: prevalence, associations, and explanation of the relationships. *J. Neuropsychiatry Clin. Neurosci.* 27, 33–41. doi: 10.1176/appi.neuropsych.13050112

Robertson, M. M., and Eapen, V. (1996). The National Hospital Interview Schedule for the assessment of Gilles de la Tourette syndrome. *Int. J. Methods Psychiatr. Res.* 6, 203–226.

Robertson, M. M., Eapen, V., and Cavanna, A. E. (2009). The international prevalence, epidemiology, and clinical phenomenology of Tourette syndrome: a cross-cultural perspective. *J. Psychosom. Res.* 67, 475–483. doi: 10.1016/j.jpsychores.2009.07.010

Robertson, M. M., Williamson, F., and Eapen, V. (2006). Depressive symptomatology in young people with Gilles de la Tourette Syndrome—A comparison of self-report scales. *J. Affect. Disord.* 91, 265–268. doi: 10.1016/j.jad.2005.12.046

Shapiro, A. K., Shapiro, E. S., Young, J. G., and Feinberg, T. E. (1988). *Gilles De La Tourette Syndrome, 2nd Edn.* New York, NY: Raven Press.

Stewart, S. E., Rosario, M. C., Baer, L., Carter, A. S., Brown, T. A., Scharf, J. M., et al. (2008). Four-factor structure of obsessive-compulsive disorder symptoms in children, adolescents, and adults. *J. Am. Acad. Child Adolesc. Psychiatry* 47, 763–772. doi: 10.1097/CHI.0b013e318172ef1e

Tanner, C. M., and Goldman, S. M. (1997). Epidemiology of Tourette syndrome. *Neurol. Clin.* 15, 395–402. doi: 10.1016/S0733-8619(05)70320-0

Trillini, M. O., and Müller-Vahl, K. R. (2015). Patients with Gilles de la Tourette syndrome have widespread personality differences. *Psychiatry Res.* 228, 765–773. doi: 10.1016/j.psychres.2015.04.043

Worbe, Y., Mallet, L., Golmard, J. L., Béhar, C., Durif, F., Jalenques, I., et al. (2010). Repetitive behaviours in patients with Gilles de la Tourette syndrome: tics, compulsions, or both. *PLoS ONE* 5:e12959. doi: 10.1371/journal.pone.0012959

Conflict of Interest Statement: The authors declare that the research was conducted in the absence of any commercial or financial relationships that could be construed as a potential conflict of interest.

Copyright © 2016 Sambrani, Jakubovski and Müller-Vahl. This is an open-access article distributed under the terms of the Creative Commons Attribution License (CC BY). The use, distribution or reproduction in other forums is permitted, provided the original author(s) or licensor are credited and that the original publication in this journal is cited, in accordance with accepted academic practice. No use, distribution or reproduction is permitted which does not comply with these terms.
